# Supplementary figures and images for: Fluorofenidone alleviates liver fibrosis by inhibiting hepatic stellate cell autophagy via the TGF-β1/Smad pathway: implications for liver cancer
Source: PeerJ. 2023 Sep 28;11:e16060. doi: 10.7717/peerj.16060 (PMC10542821; doi:10.7717/peerj.16060)

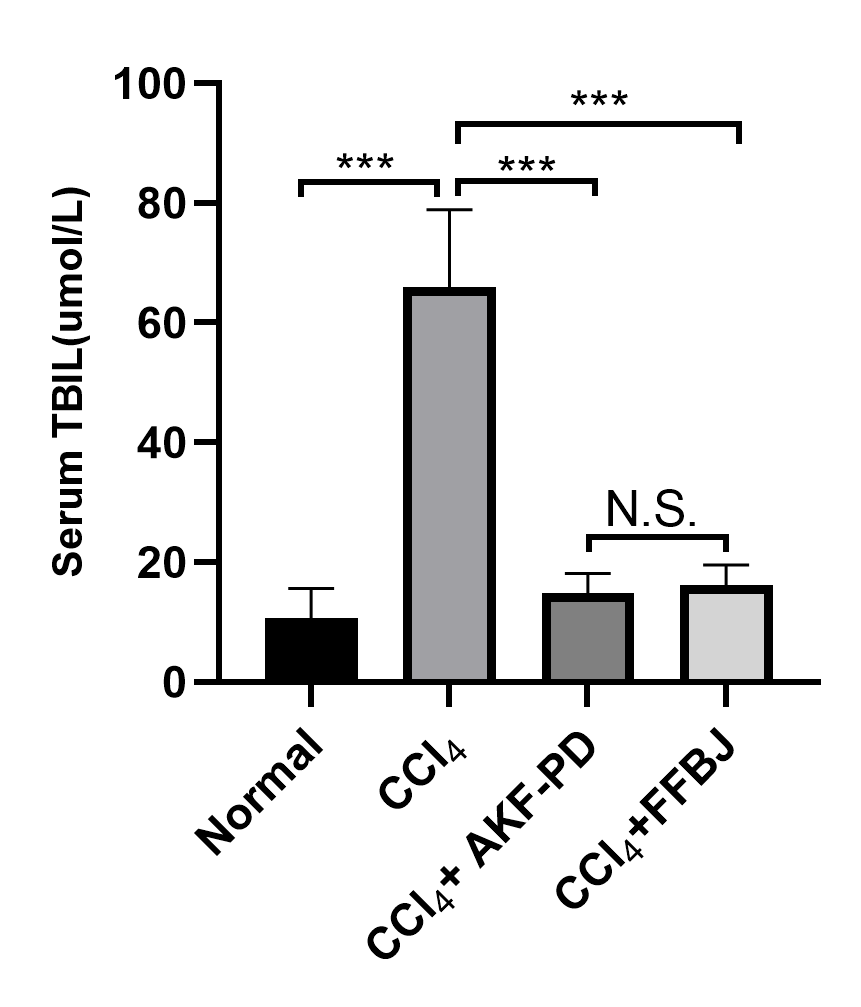

Supplement: Supplemental Information 1 [file peerj-11-16060-s001.zip › Fig1/Fig1C/Serum TBIL(umol_L).tif]

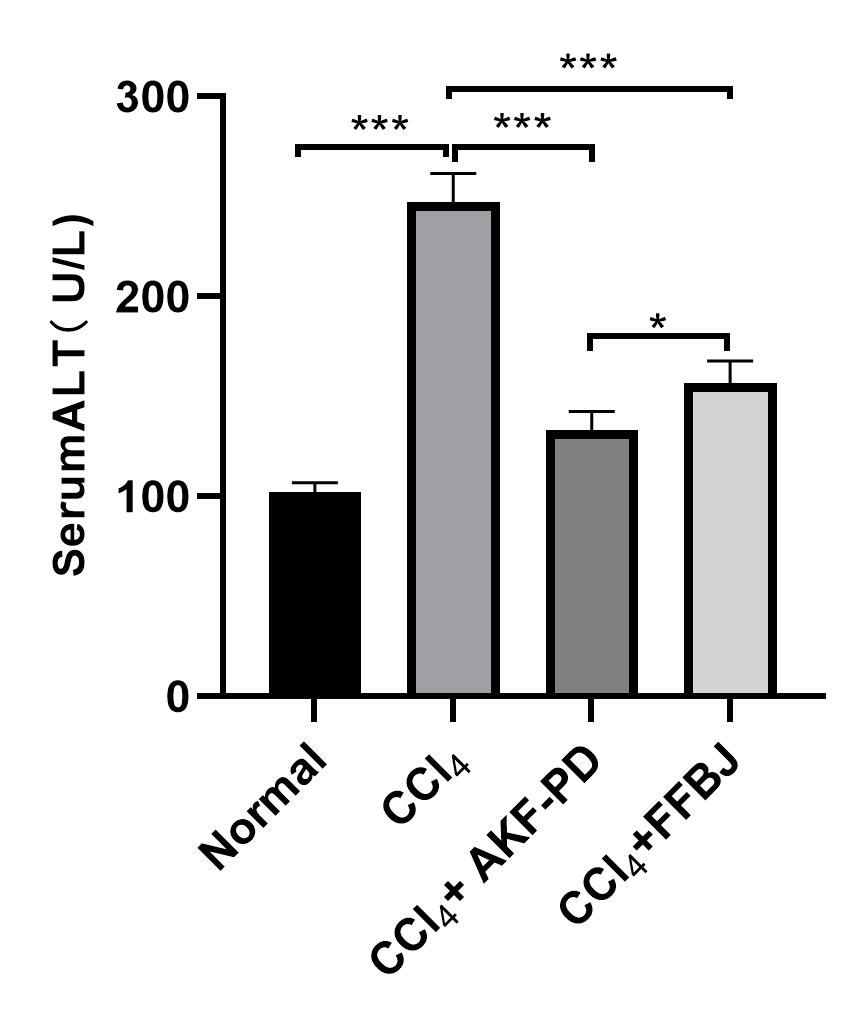

Supplement: Supplemental Information 1 [file peerj-11-16060-s001.zip › Fig1/Fig1C/SerumALT∩╝êU_L).tif]

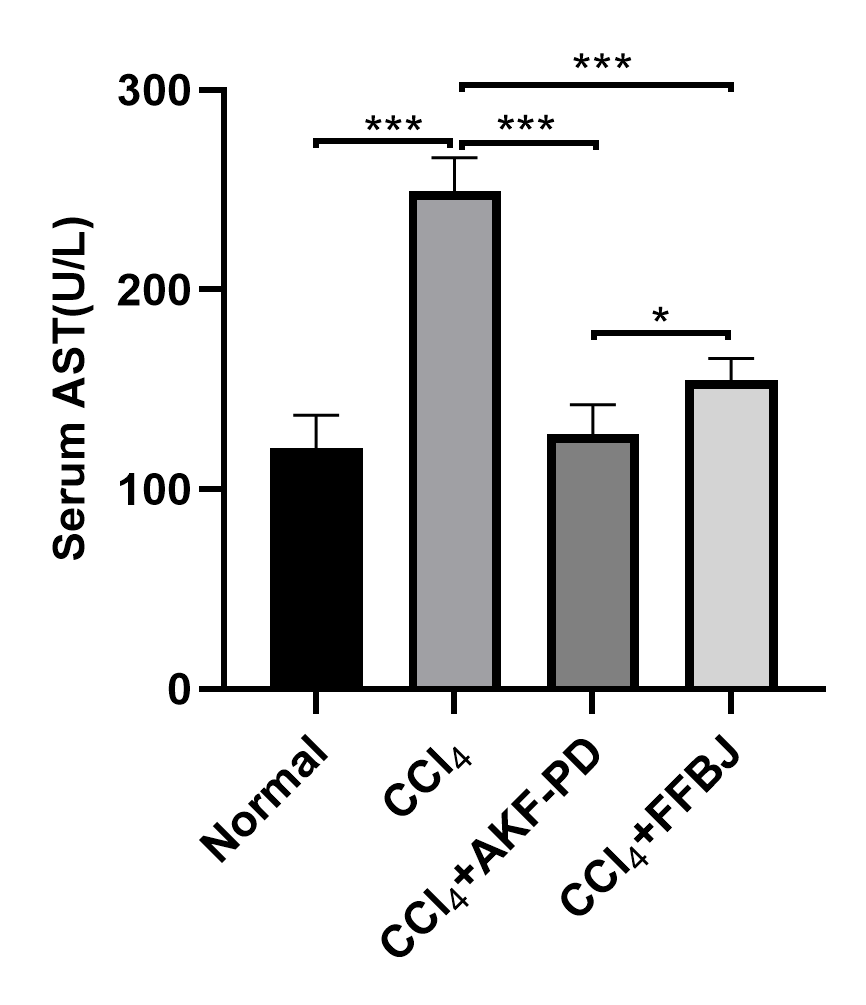

Supplement: Supplemental Information 1 [file peerj-11-16060-s001.zip › Fig1/Fig1C/Serum AST(U_L).tif]

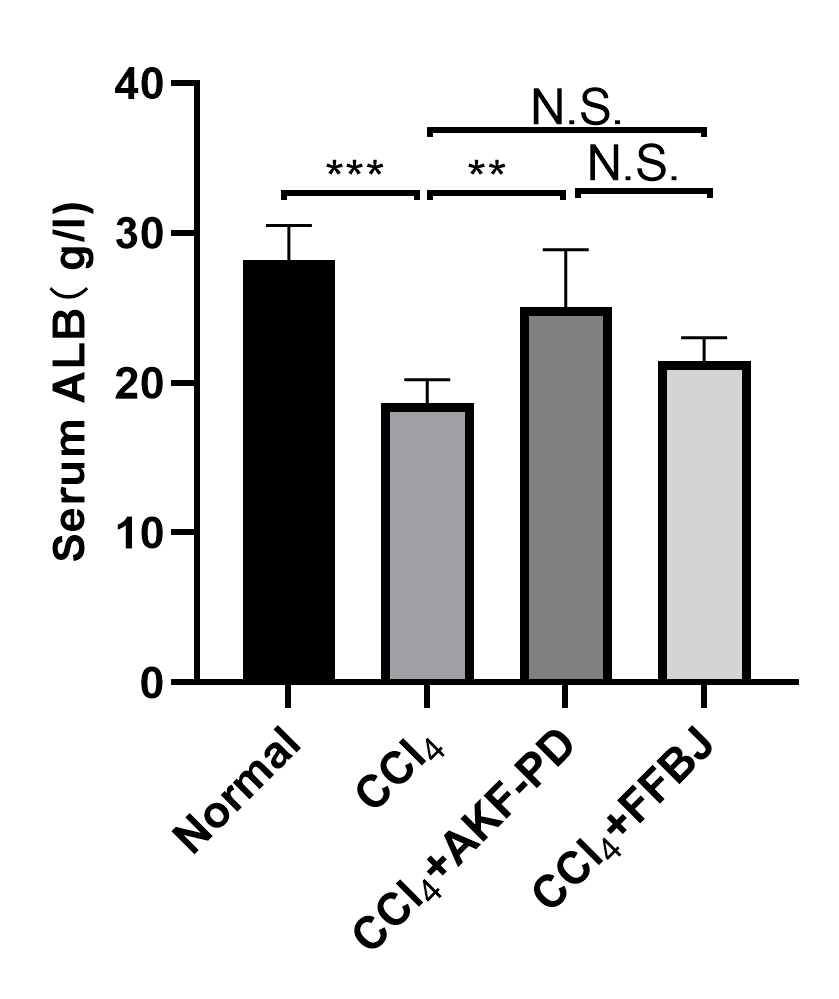

Supplement: Supplemental Information 1 [file peerj-11-16060-s001.zip › Fig1/Fig1C/Serum ALB∩╝êg_l).tif]

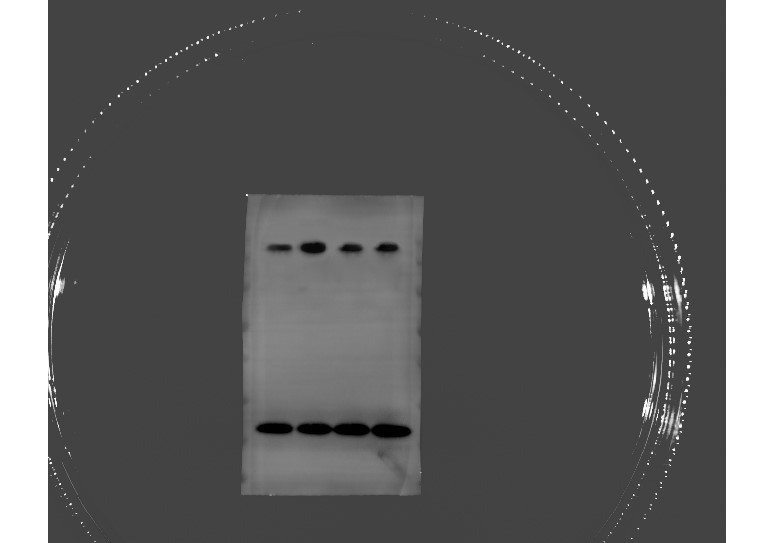

Supplement: Supplemental Information 1 [file peerj-11-16060-s001.zip › Fig1/Fig1B/CollagenIII∩╝êΣ╕èCollagenIIIπÇüΣ╕ïGAPDH∩╝ë .jpg]

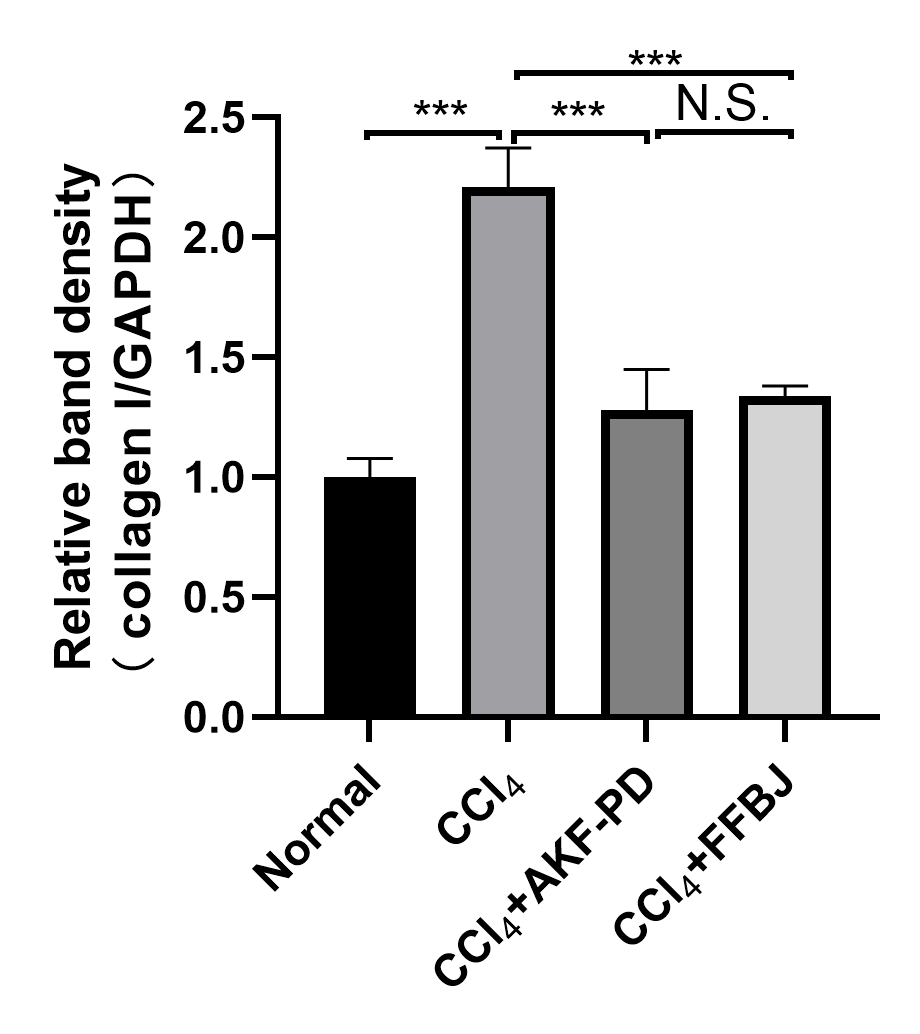

Supplement: Supplemental Information 1 [file peerj-11-16060-s001.zip › Fig1/Fig1B/Relative band density∩╝êcollagen I_GAPDH∩╝ë.tif]

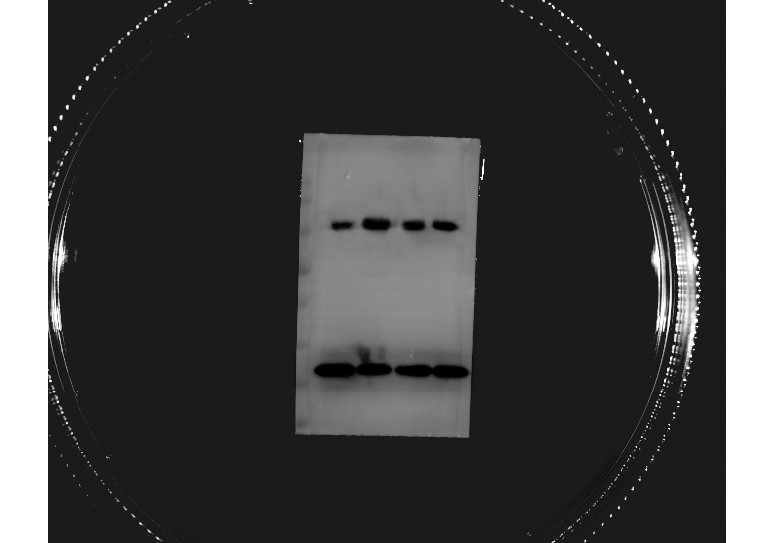

Supplement: Supplemental Information 1 [file peerj-11-16060-s001.zip › Fig1/Fig1B/CollagenI∩╝êΣ╕è∩╝ë .jpg]

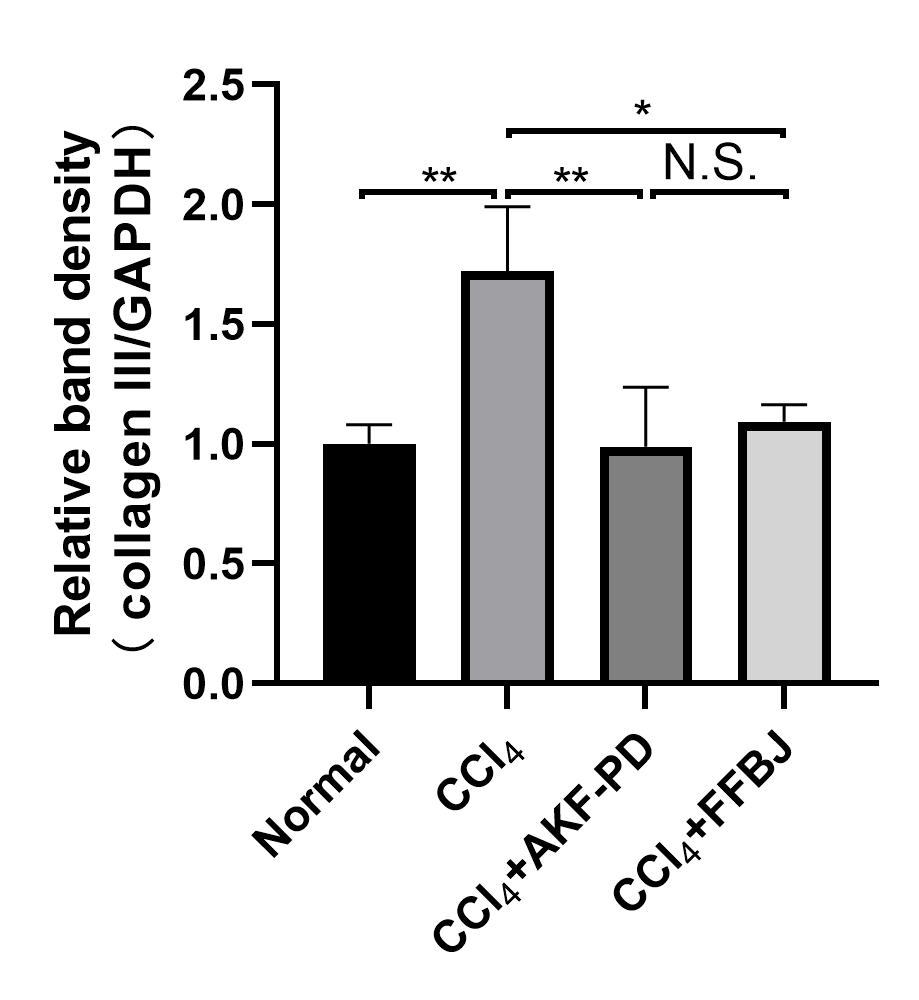

Supplement: Supplemental Information 1 [file peerj-11-16060-s001.zip › Fig1/Fig1B/Relative band density∩╝êcollagen III_GAPDH∩╝ë.tif]

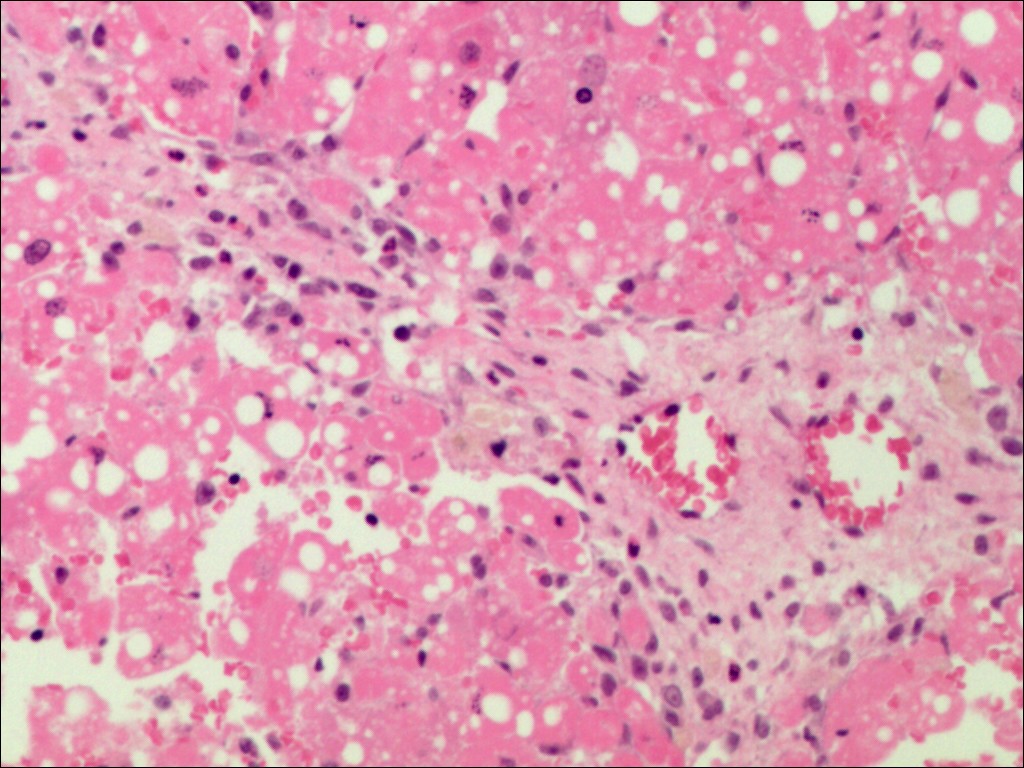

Supplement: Supplemental Information 1 [file peerj-11-16060-s001.zip › Fig1/Fig1A/H&E staining/CCl4 group.jpg]

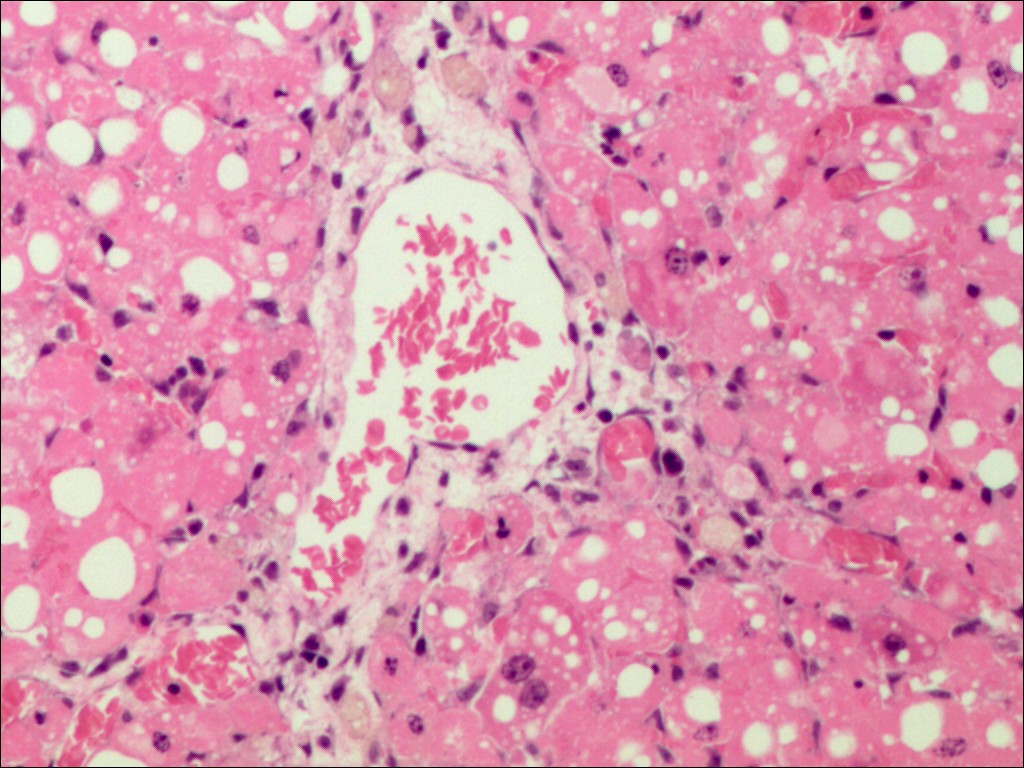

Supplement: Supplemental Information 1 [file peerj-11-16060-s001.zip › Fig1/Fig1A/H&E staining/CCl4+FFBJ group.jpg]

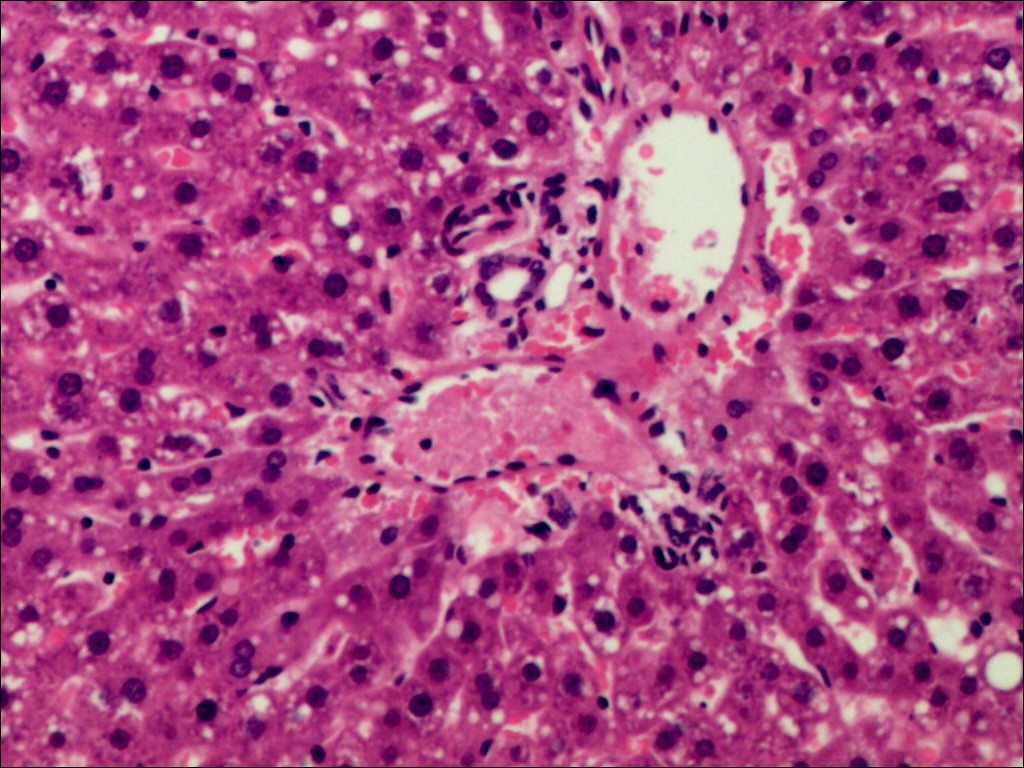

Supplement: Supplemental Information 1 [file peerj-11-16060-s001.zip › Fig1/Fig1A/H&E staining/CCl4+AKF-PD group.jpg]

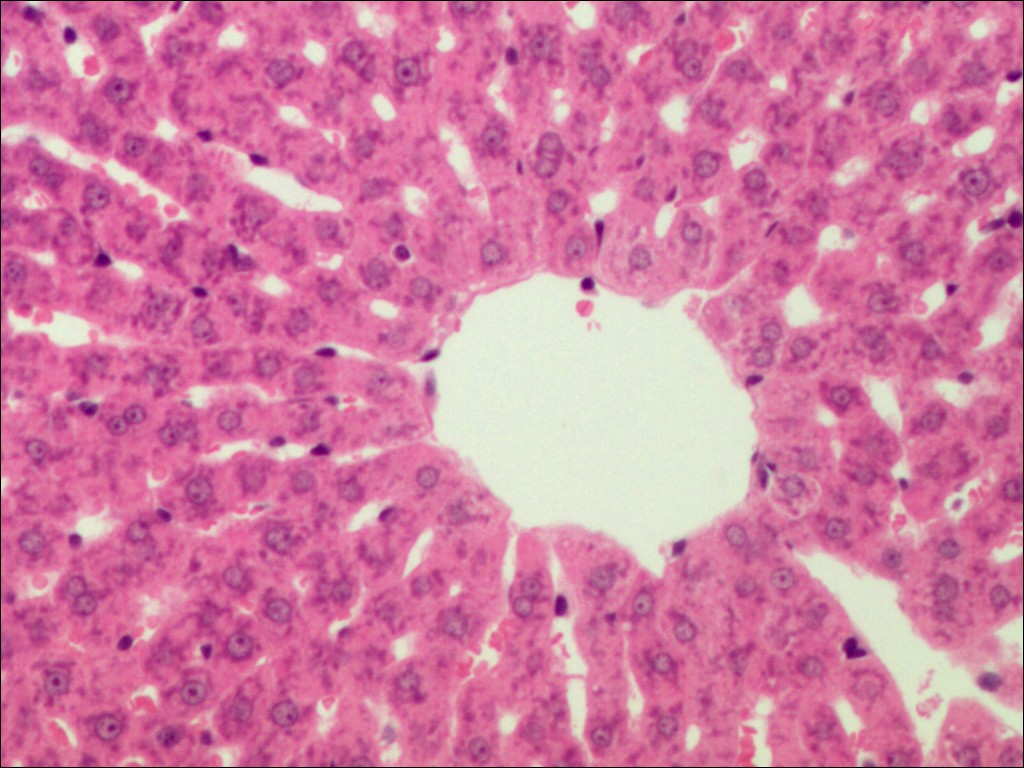

Supplement: Supplemental Information 1 [file peerj-11-16060-s001.zip › Fig1/Fig1A/H&E staining/Normal group.jpg]

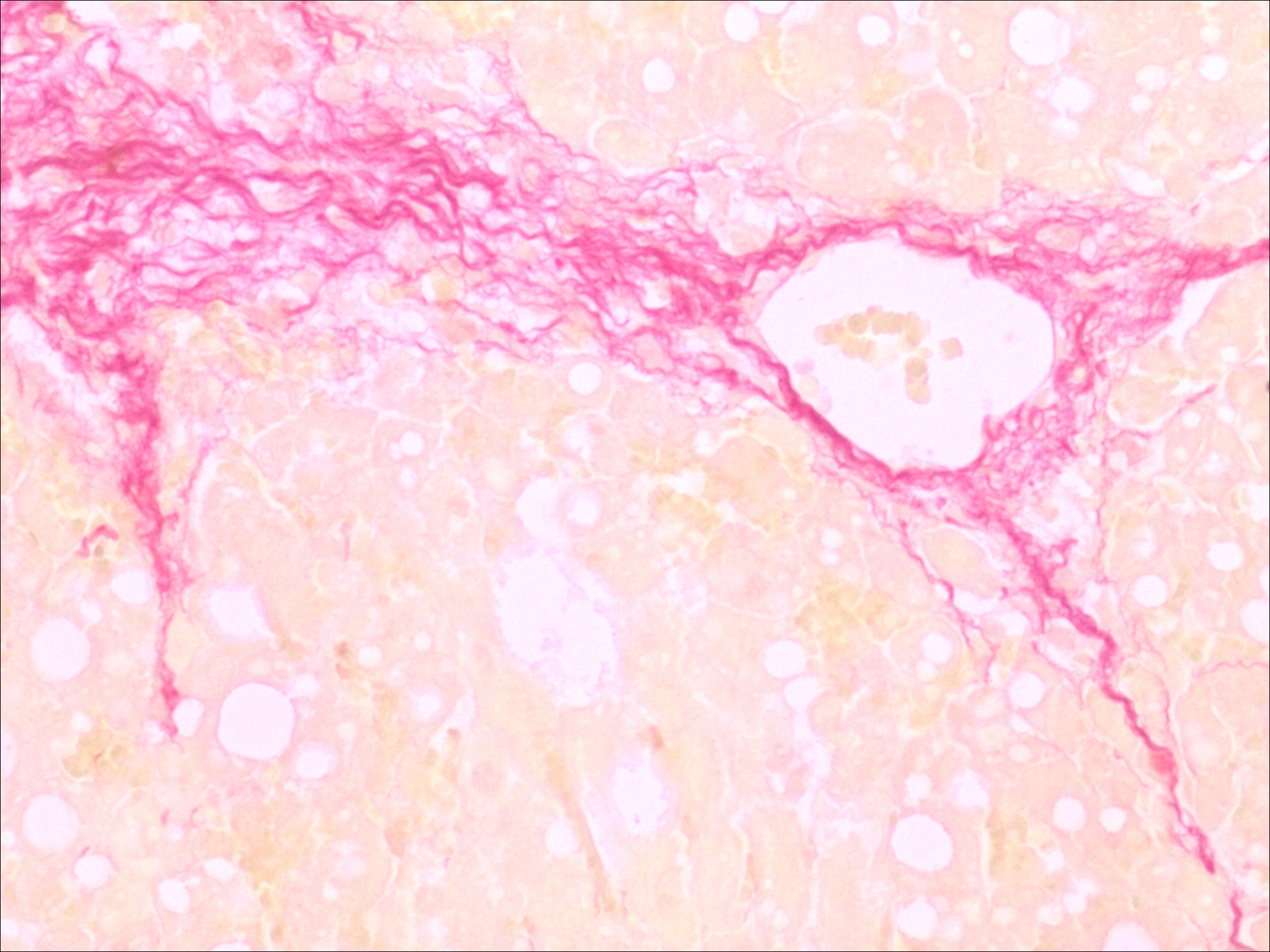

Supplement: Supplemental Information 1 [file peerj-11-16060-s001.zip › Fig1/Fig1A/Sirius Red staining/CCl4 group.jpg]

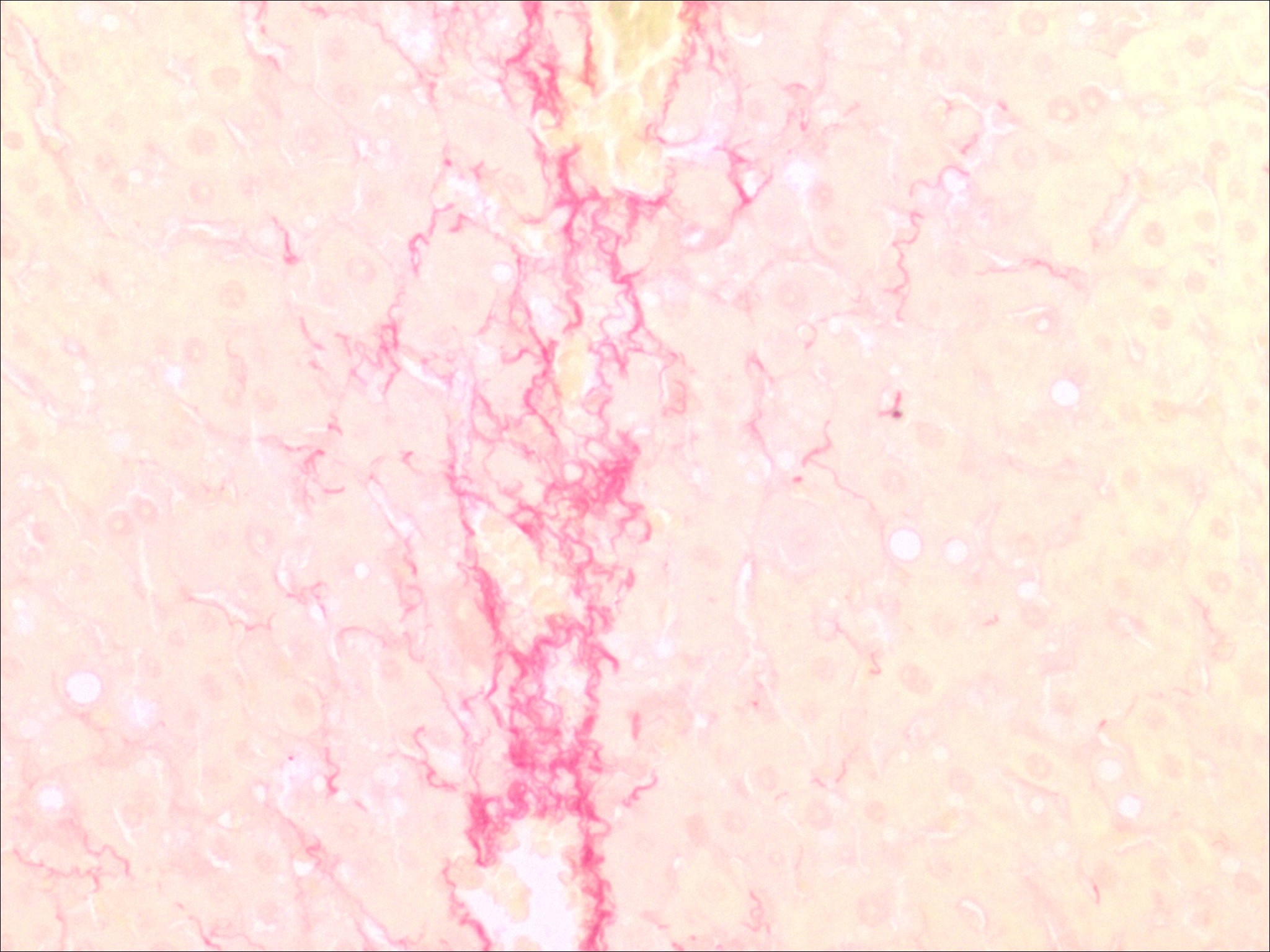

Supplement: Supplemental Information 1 [file peerj-11-16060-s001.zip › Fig1/Fig1A/Sirius Red staining/CCl4+FFBJ group.jpg]

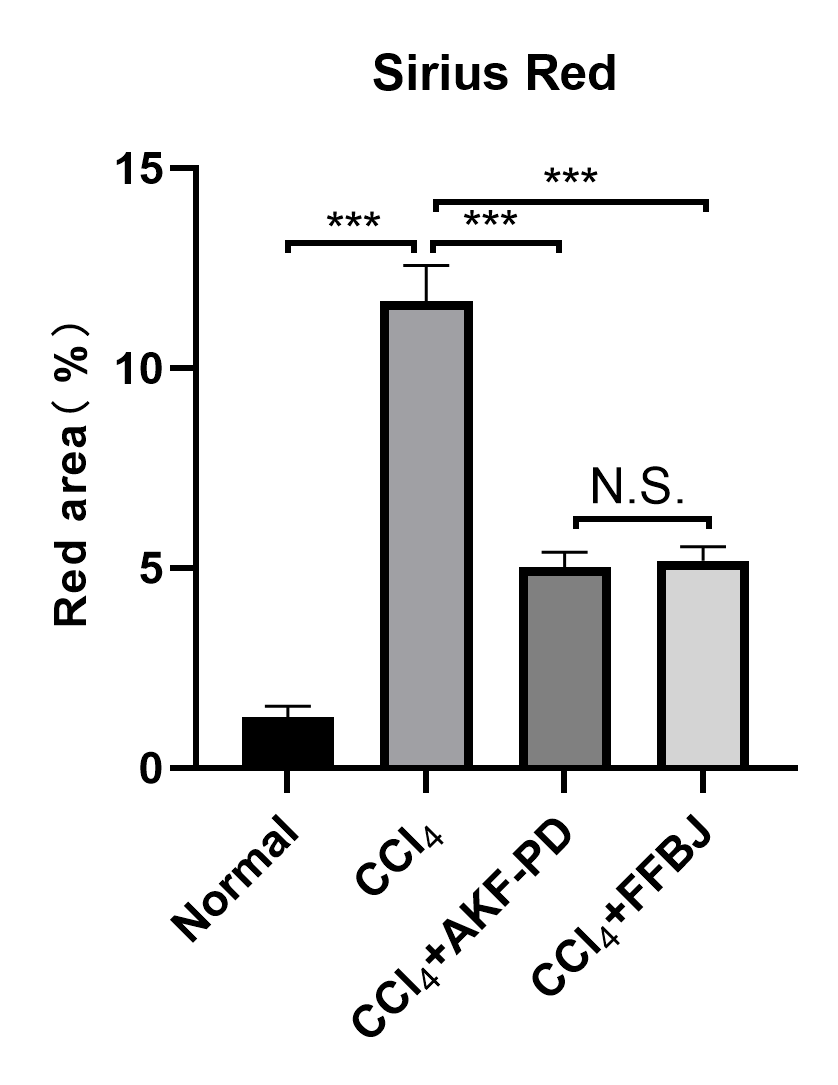

Supplement: Supplemental Information 1 [file peerj-11-16060-s001.zip › Fig1/Fig1A/Sirius Red staining/Red area∩╝ê%∩╝ë.tif]

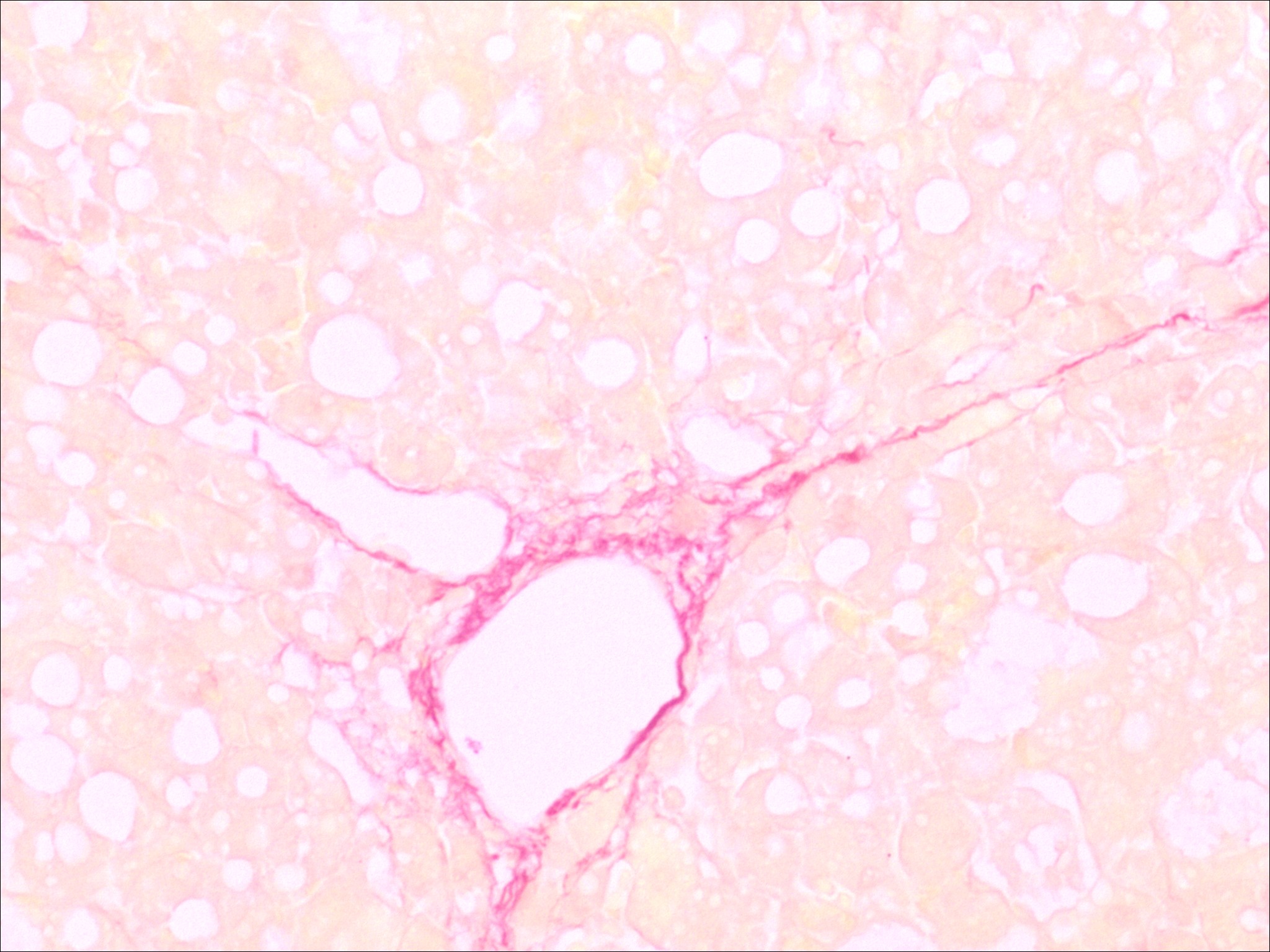

Supplement: Supplemental Information 1 [file peerj-11-16060-s001.zip › Fig1/Fig1A/Sirius Red staining/CCl4+AKF-PD group.jpg]

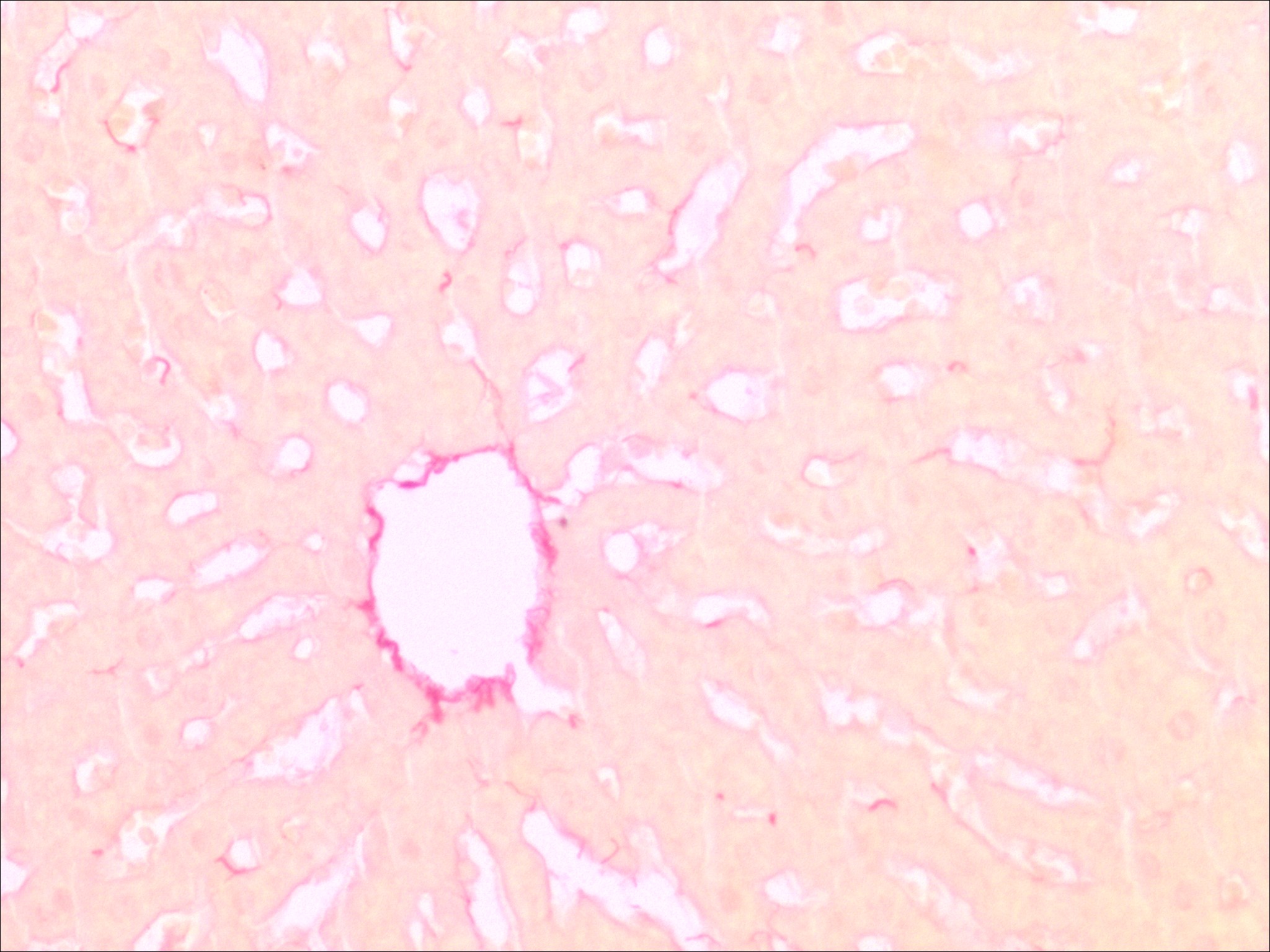

Supplement: Supplemental Information 1 [file peerj-11-16060-s001.zip › Fig1/Fig1A/Sirius Red staining/Normal group.jpg]

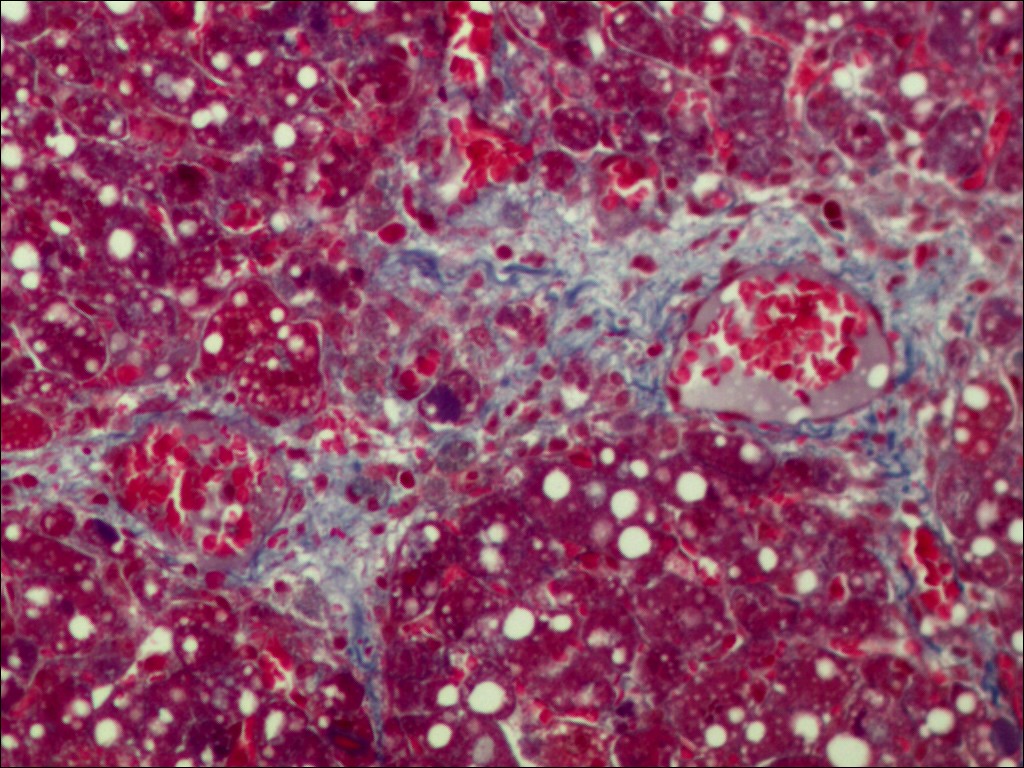

Supplement: Supplemental Information 1 [file peerj-11-16060-s001.zip › Fig1/Fig1A/Masson staining/CCl4 group.jpg]

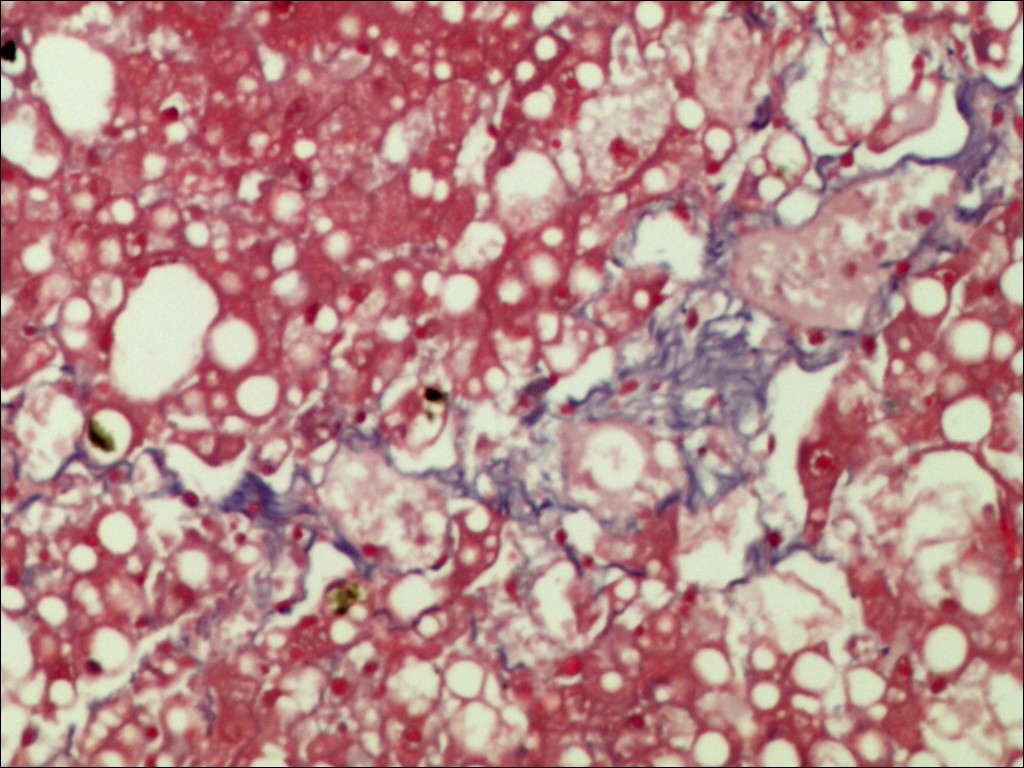

Supplement: Supplemental Information 1 [file peerj-11-16060-s001.zip › Fig1/Fig1A/Masson staining/CCl4+FFBJ group.jpg]

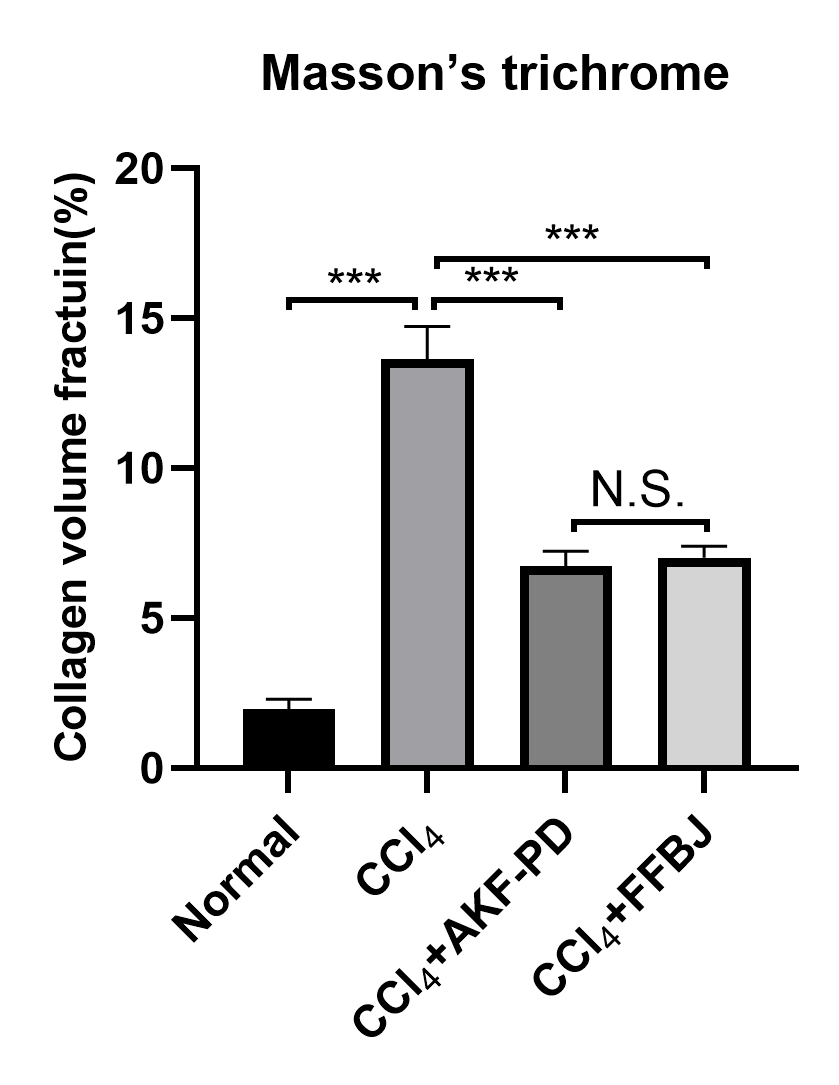

Supplement: Supplemental Information 1 [file peerj-11-16060-s001.zip › Fig1/Fig1A/Masson staining/MassonΓÇÖs trichrome positive area(%).tif]

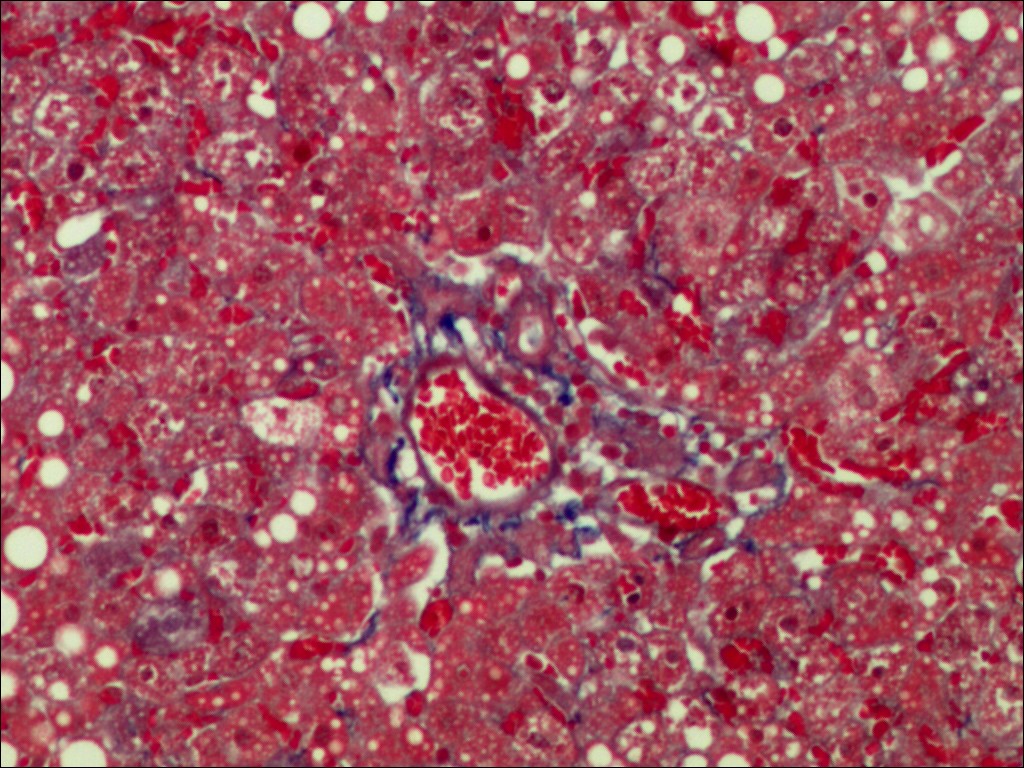

Supplement: Supplemental Information 1 [file peerj-11-16060-s001.zip › Fig1/Fig1A/Masson staining/CCl4+AKF-PD group.jpg]

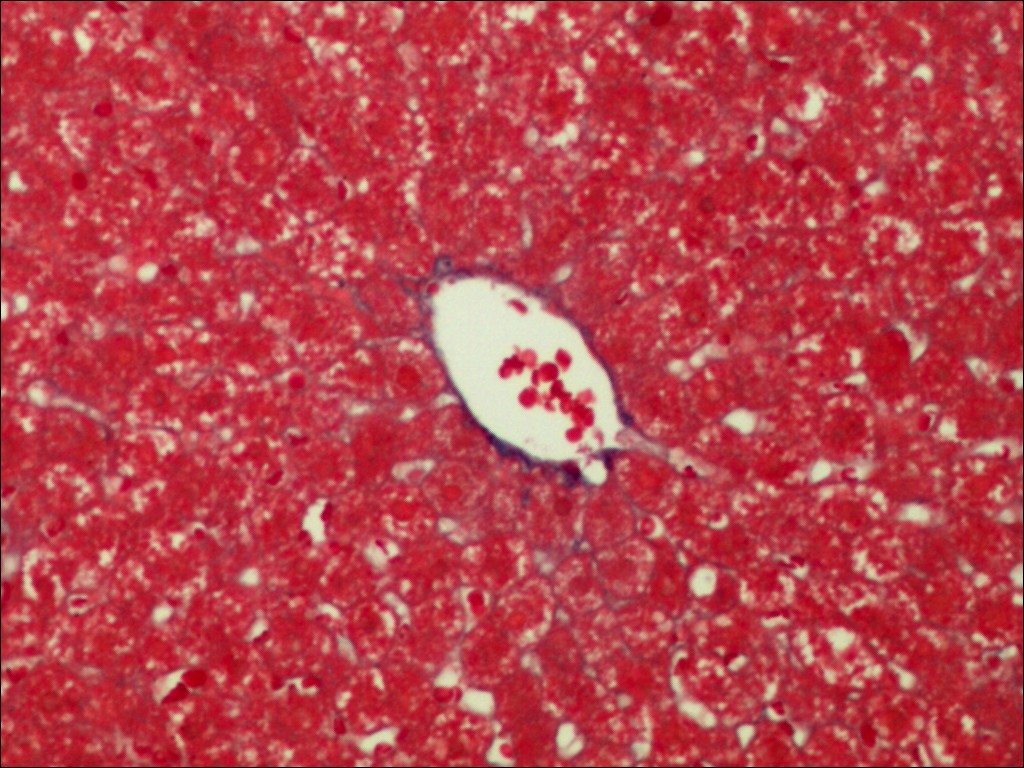

Supplement: Supplemental Information 1 [file peerj-11-16060-s001.zip › Fig1/Fig1A/Masson staining/Normal group.jpg]

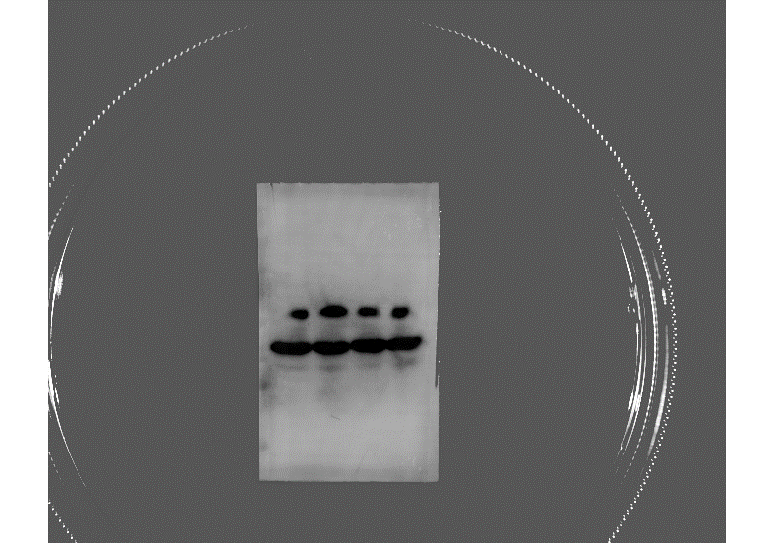

Supplement: Supplemental Information 2 [file peerj-11-16060-s002.zip › Fig2/Fig2B/Beclin-1∩╝êΣ╕èBeclin-1πÇüΣ╕ïGAPDH).png]

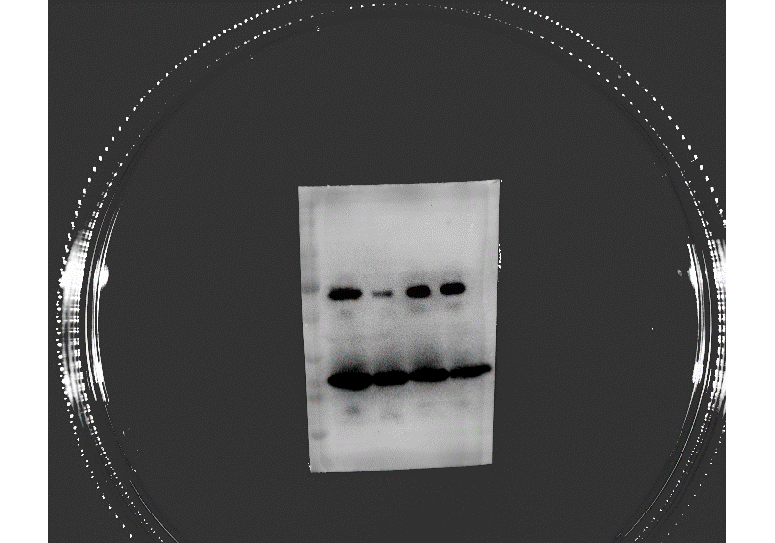

Supplement: Supplemental Information 2 [file peerj-11-16060-s002.zip › Fig2/Fig2B/P62∩╝êΣ╕è∩╝ë.png]

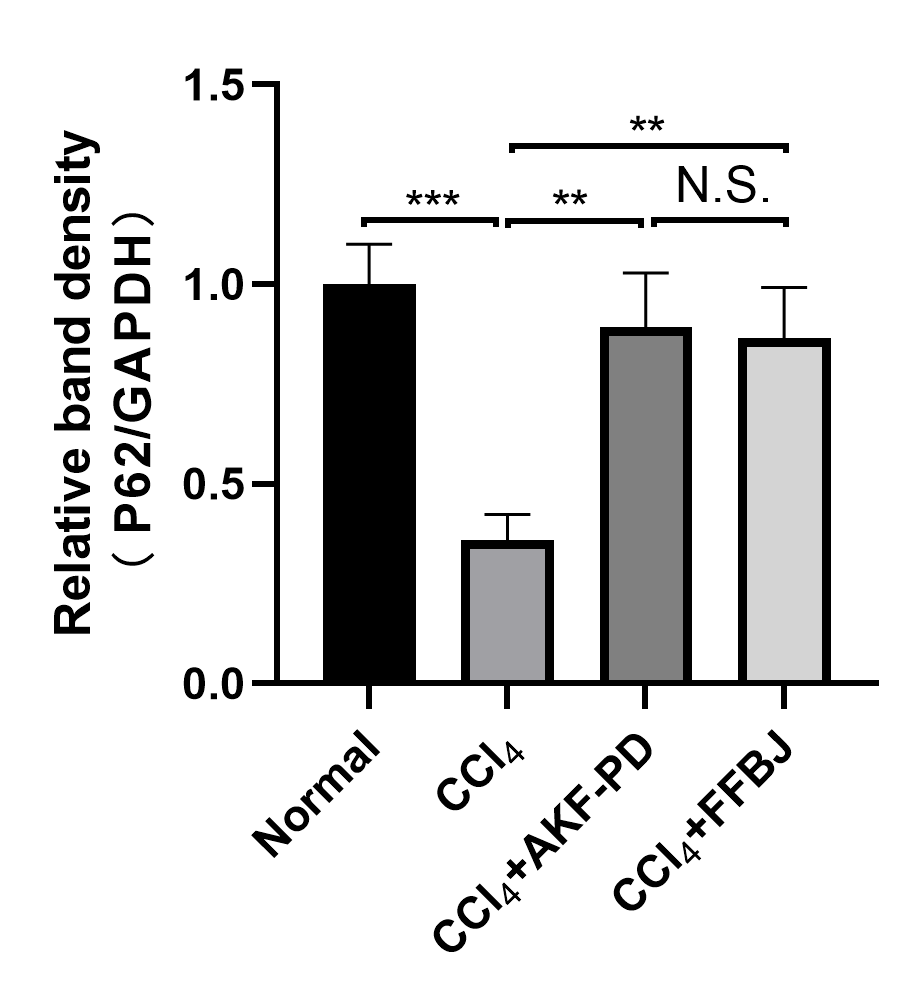

Supplement: Supplemental Information 2 [file peerj-11-16060-s002.zip › Fig2/Fig2B/Relative band density∩╝êP62_GAPDH∩╝ë.tif]

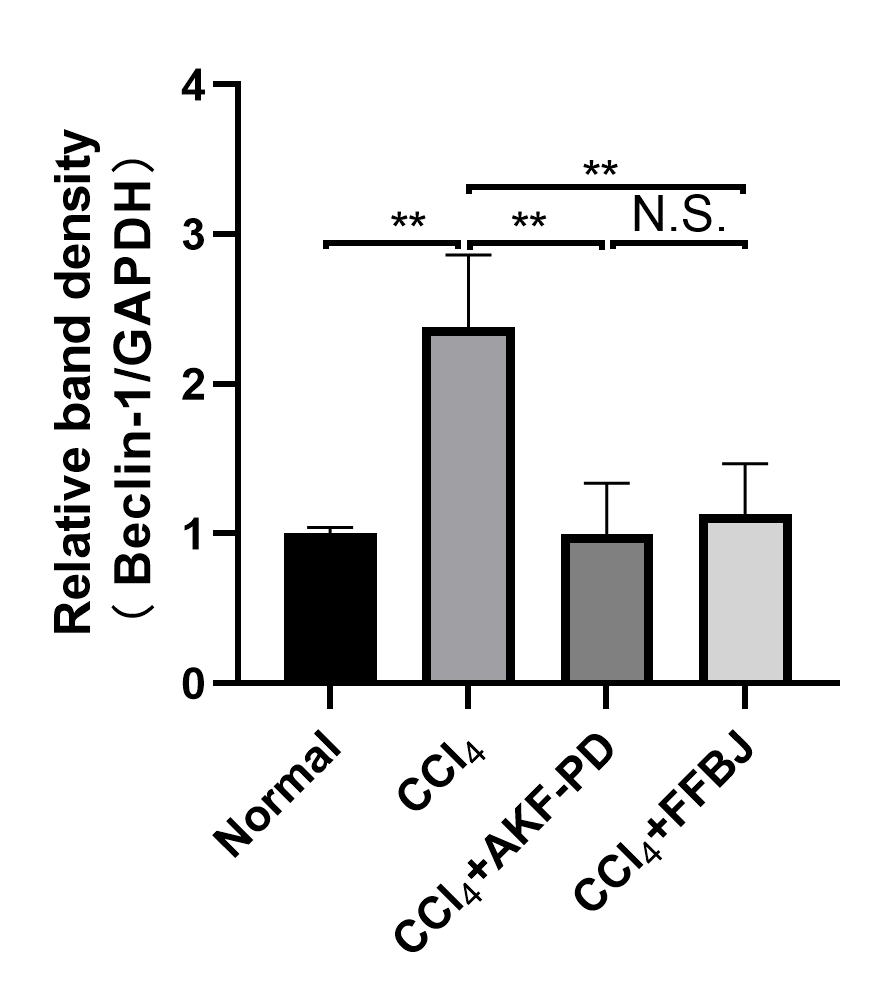

Supplement: Supplemental Information 2 [file peerj-11-16060-s002.zip › Fig2/Fig2B/Relative band density∩╝êBeclin-1_GAPDH∩╝ë.tif]

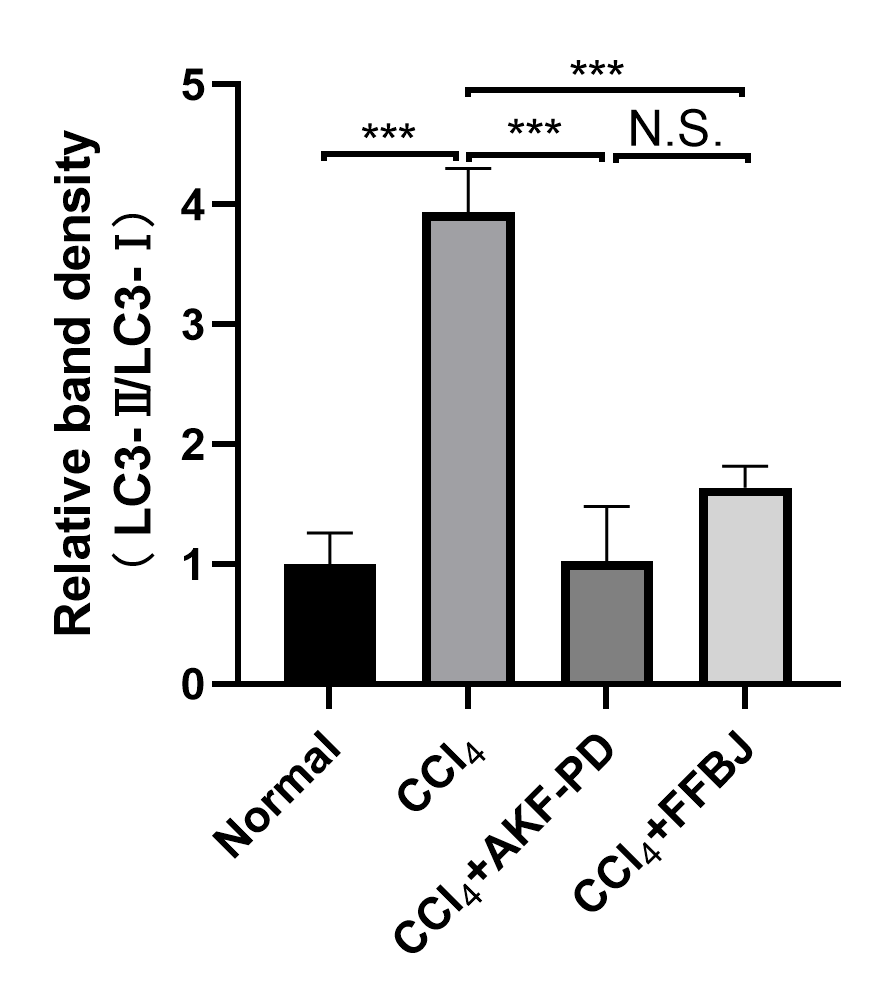

Supplement: Supplemental Information 2 [file peerj-11-16060-s002.zip › Fig2/Fig2B/Relative band density∩╝êLC3-II_LC3-Γàá∩╝ë.tif]

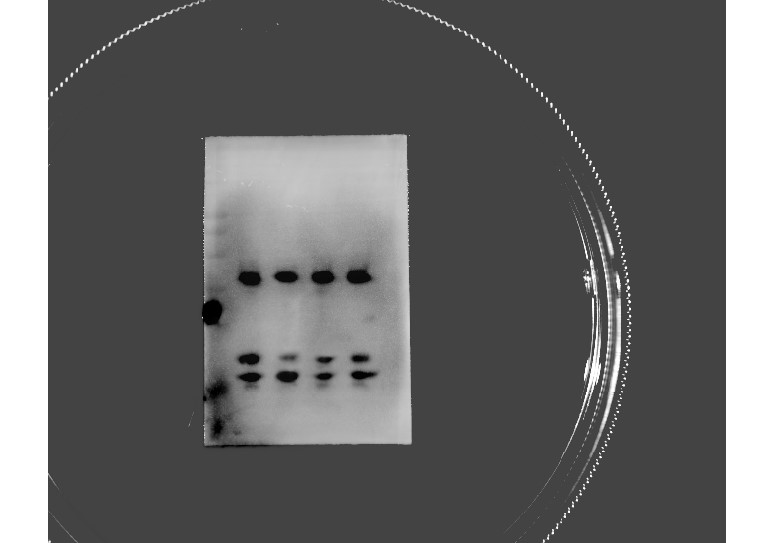

Supplement: Supplemental Information 2 [file peerj-11-16060-s002.zip › Fig2/Fig2B/LC3∩╝êΣ╕èGAPDHπÇüΣ╕ïLC3-IσÆîLC3-II).jpg]

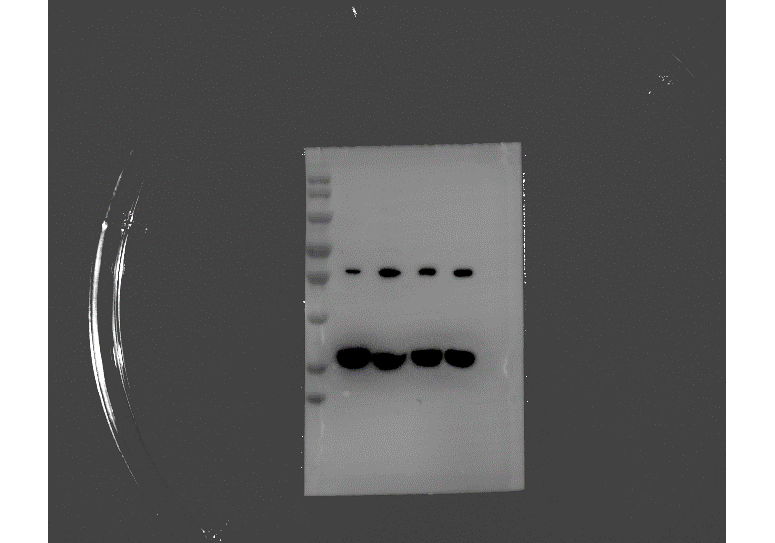

Supplement: Supplemental Information 2 [file peerj-11-16060-s002.zip › Fig2/Fig2A/p-Smad2∩╝êΣ╕è∩╝ë.png]

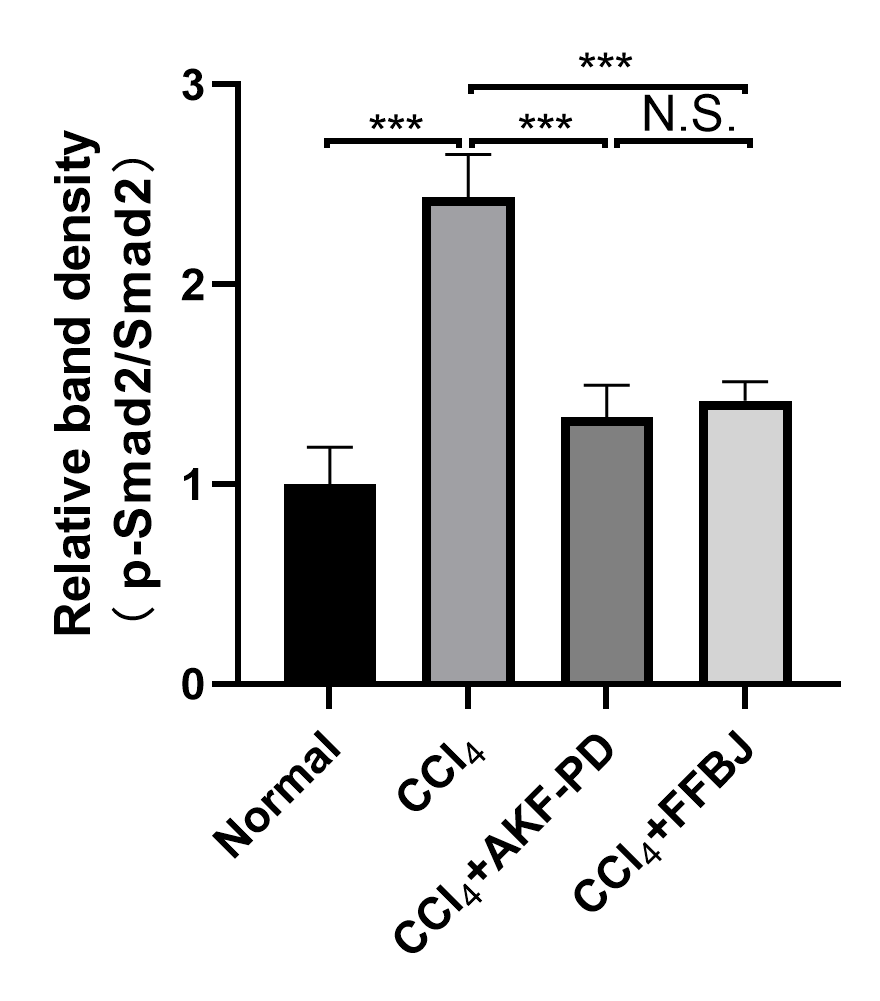

Supplement: Supplemental Information 2 [file peerj-11-16060-s002.zip › Fig2/Fig2A/Relative band density∩╝êp-Smad2_Smad2∩╝ë.tif]

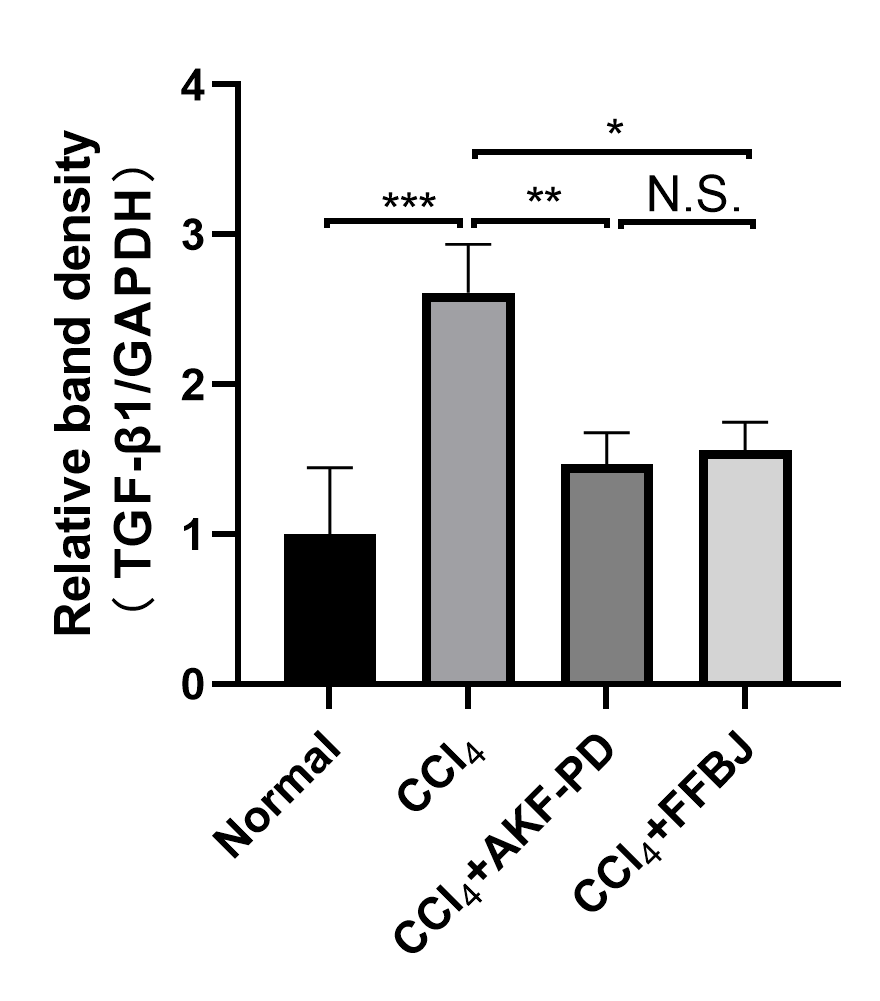

Supplement: Supplemental Information 2 [file peerj-11-16060-s002.zip › Fig2/Fig2A/Relative band density∩╝êTGF-╬▓1_GAPDH∩╝ë.tif]

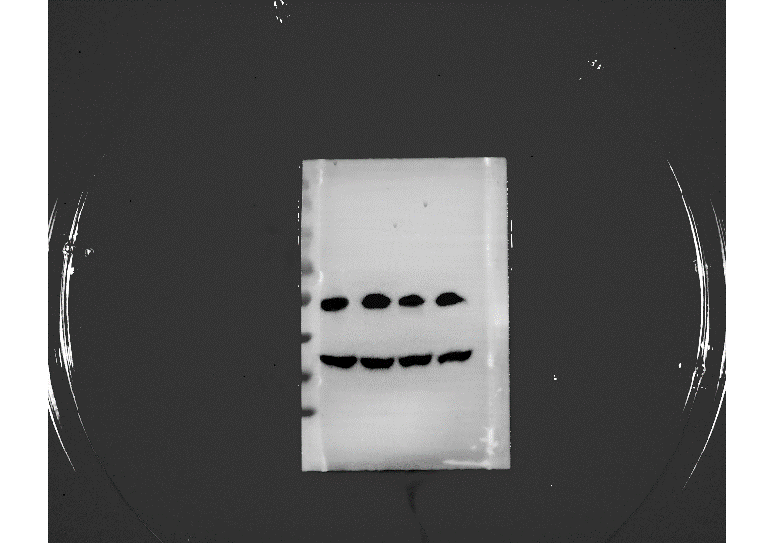

Supplement: Supplemental Information 2 [file peerj-11-16060-s002.zip › Fig2/Fig2A/Smad3∩╝êΣ╕è∩╝ë .png]

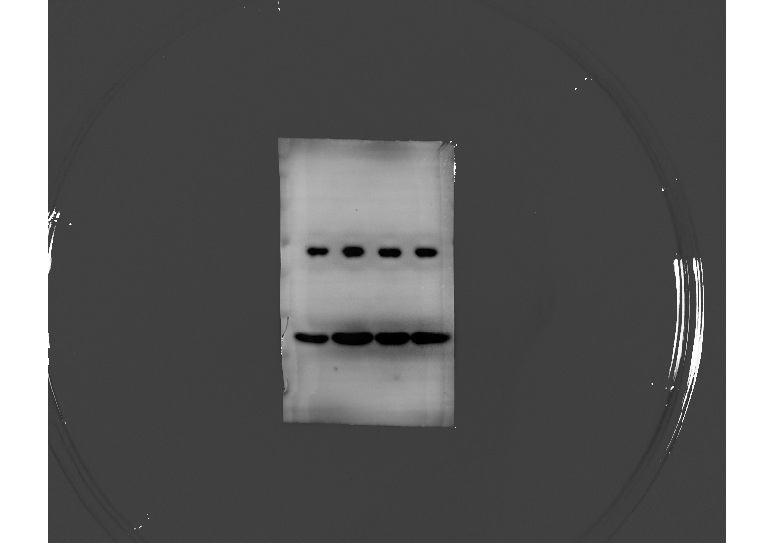

Supplement: Supplemental Information 2 [file peerj-11-16060-s002.zip › Fig2/Fig2A/Smad2∩╝êΣ╕è∩╝ë .png]

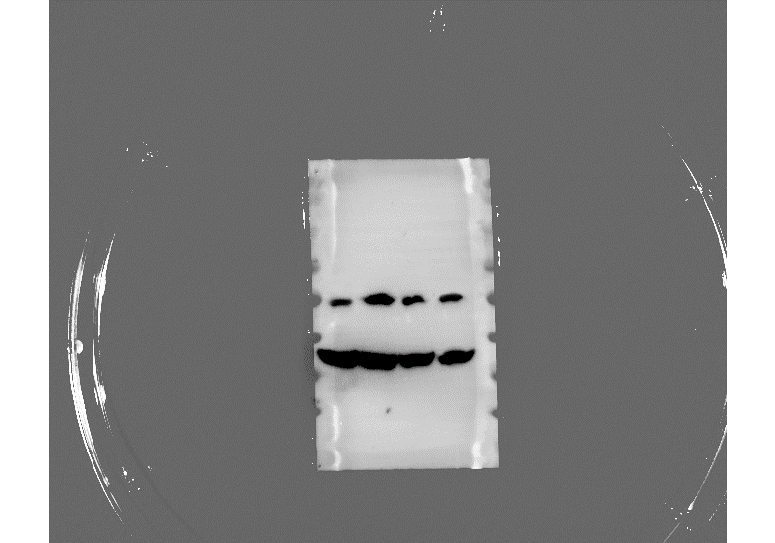

Supplement: Supplemental Information 2 [file peerj-11-16060-s002.zip › Fig2/Fig2A/p-Smad3∩╝êΣ╕è∩╝ë .png]

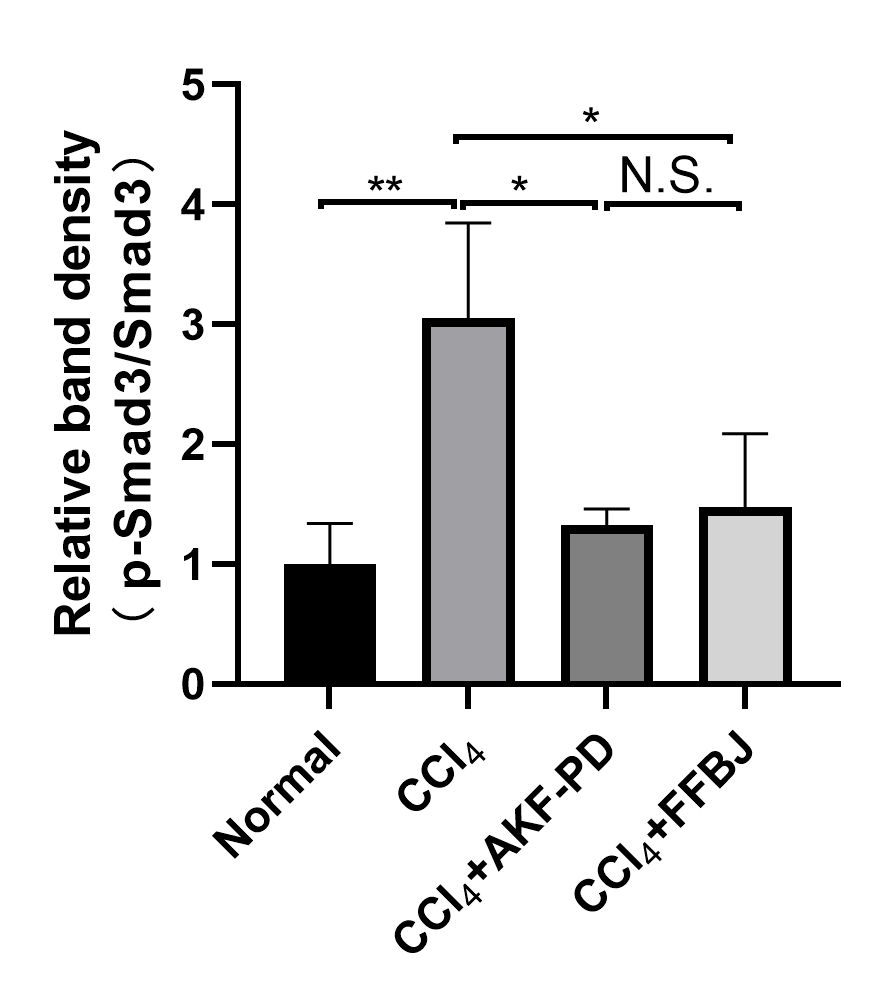

Supplement: Supplemental Information 2 [file peerj-11-16060-s002.zip › Fig2/Fig2A/Relative band density∩╝êp-Smad3_Smad3∩╝ë.tif]

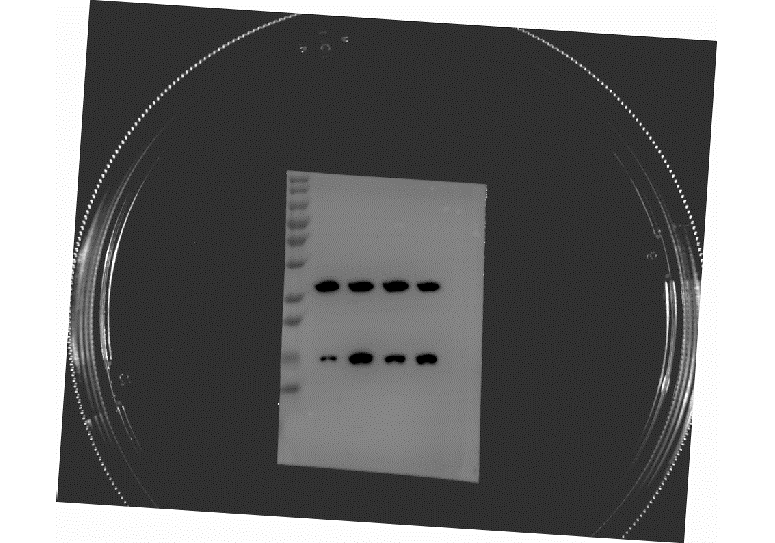

Supplement: Supplemental Information 2 [file peerj-11-16060-s002.zip › Fig2/Fig2A/TGF-╬▓ 1(Σ╕èGAPDHπÇüΣ╕ïTGF-╬▓1∩╝ë.png]

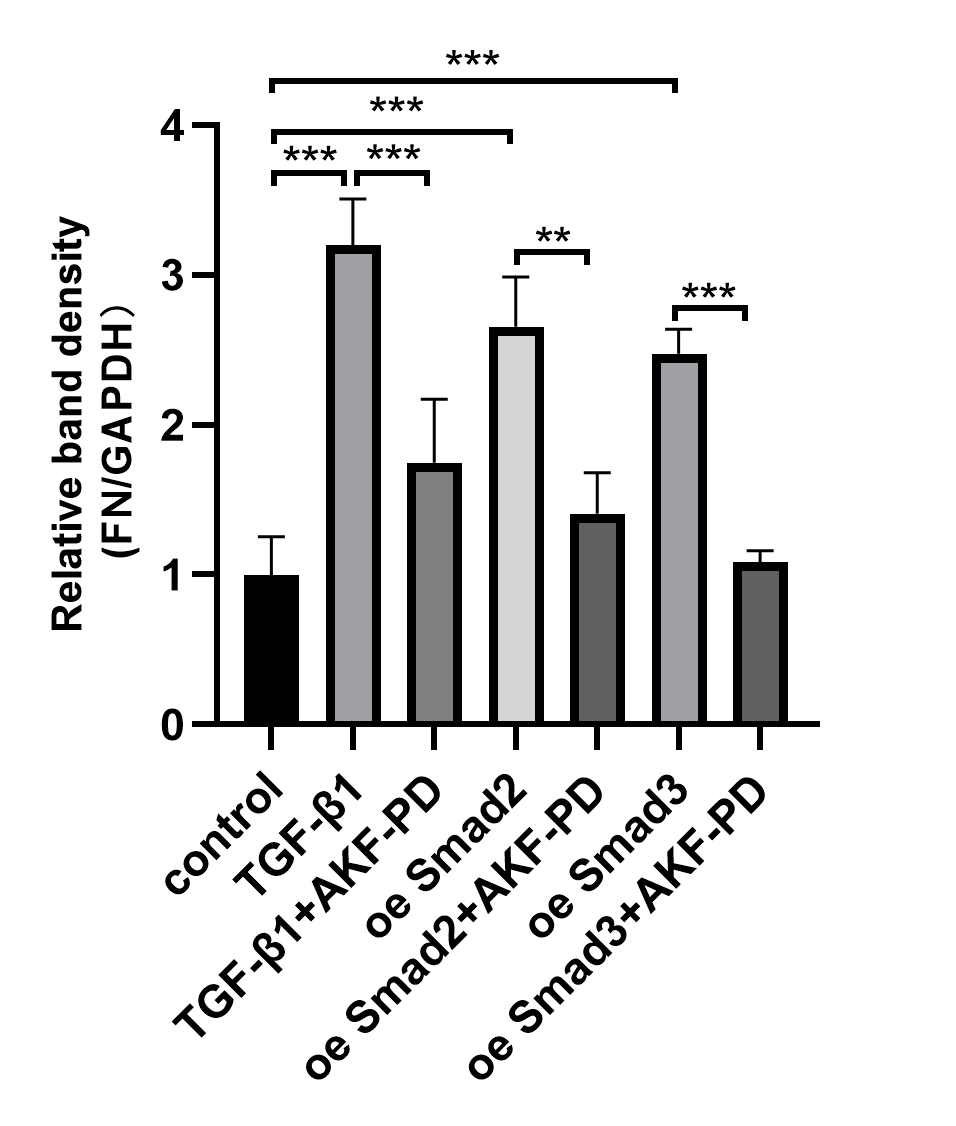

Supplement: Supplemental Information 3 [file peerj-11-16060-s003.zip › Fig3/Relative band density(FN_GAPDH∩╝ë.tif]

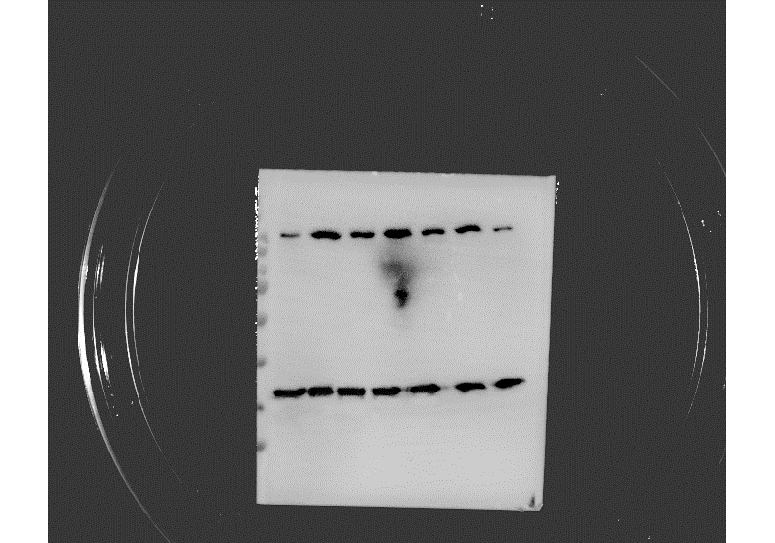

Supplement: Supplemental Information 3 [file peerj-11-16060-s003.zip › Fig3/FN∩╝êΣ╕è∩╝ë.png]

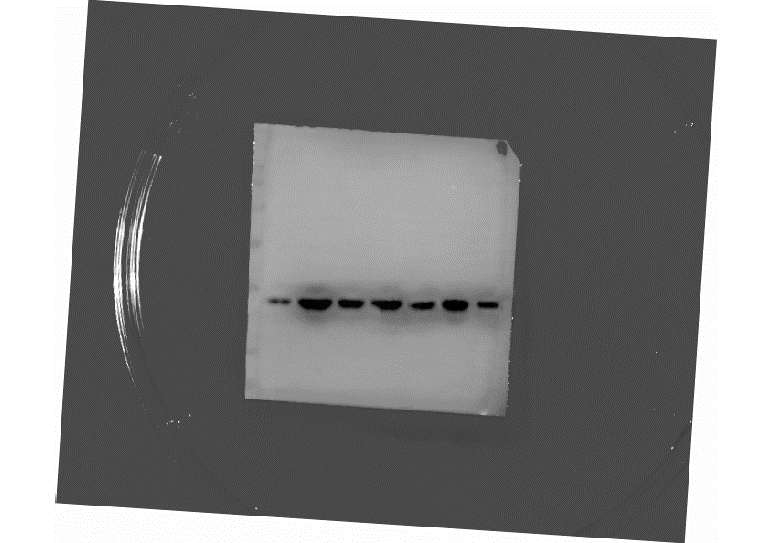

Supplement: Supplemental Information 3 [file peerj-11-16060-s003.zip › Fig3/a-SMA .png]

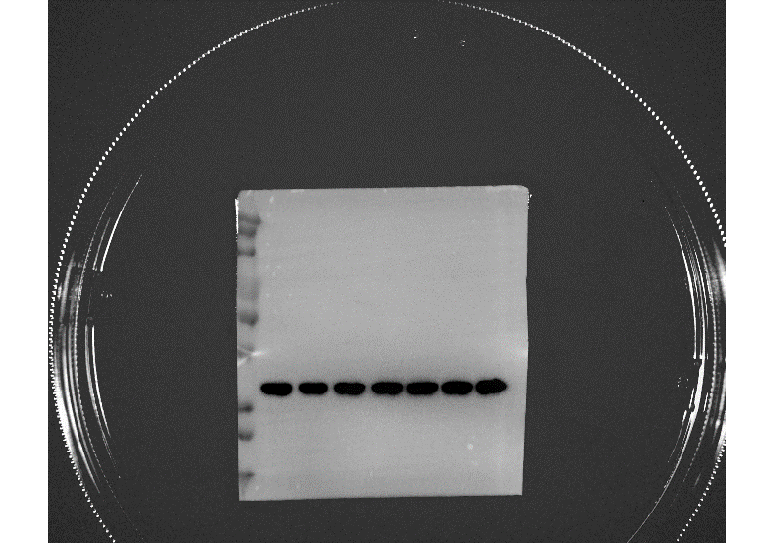

Supplement: Supplemental Information 3 [file peerj-11-16060-s003.zip › Fig3/GAPDH.png]

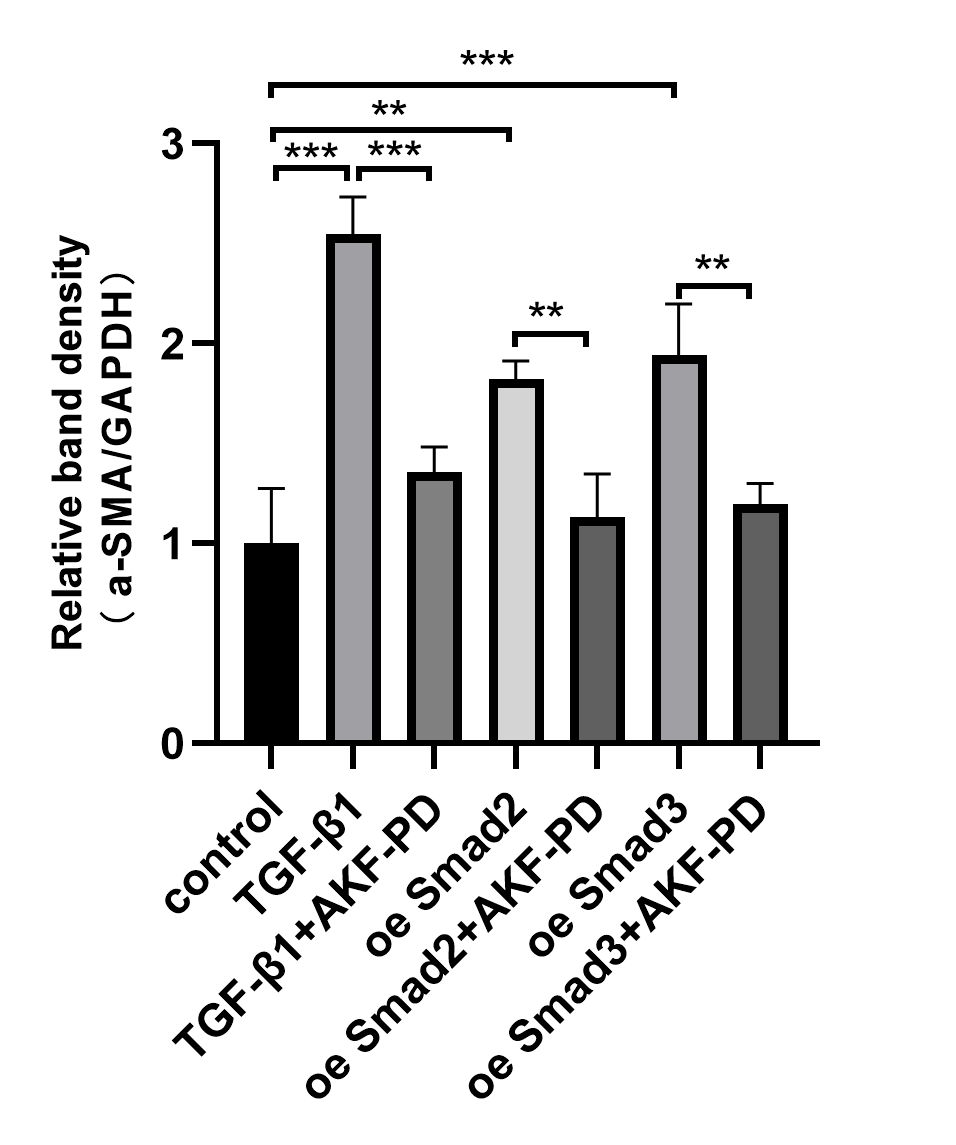

Supplement: Supplemental Information 3 [file peerj-11-16060-s003.zip › Fig3/Relative band density∩╝êa-SMA_GAPDH∩╝ë.tif]

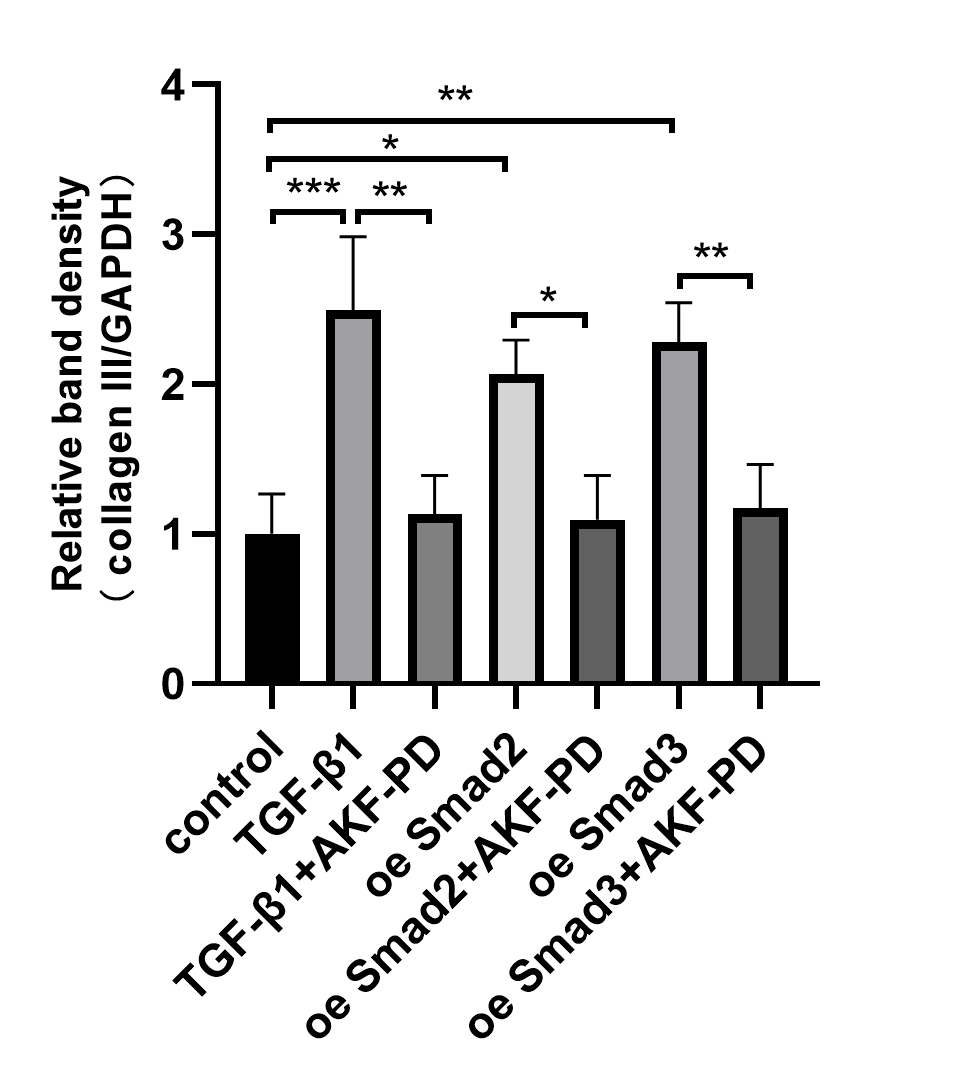

Supplement: Supplemental Information 3 [file peerj-11-16060-s003.zip › Fig3/Relative band density∩╝êcollagen III_GAPDH∩╝ë.tif]

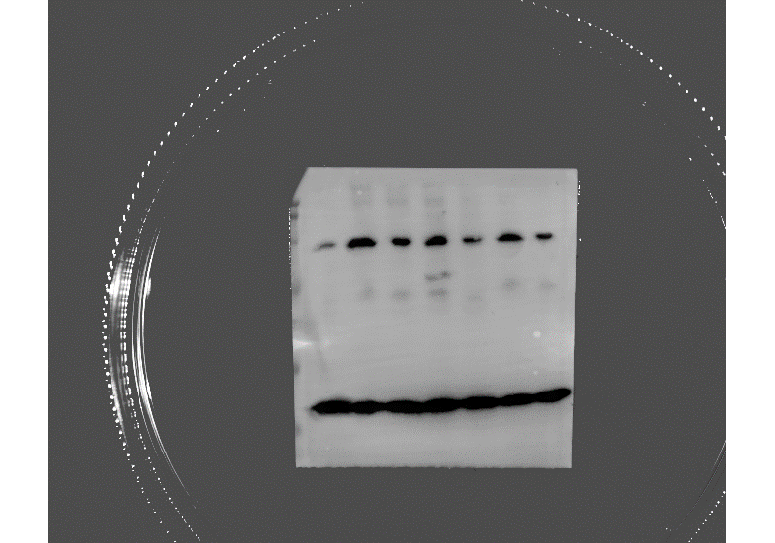

Supplement: Supplemental Information 3 [file peerj-11-16060-s003.zip › Fig3/collagen III∩╝êΣ╕è∩╝ë 3.png]

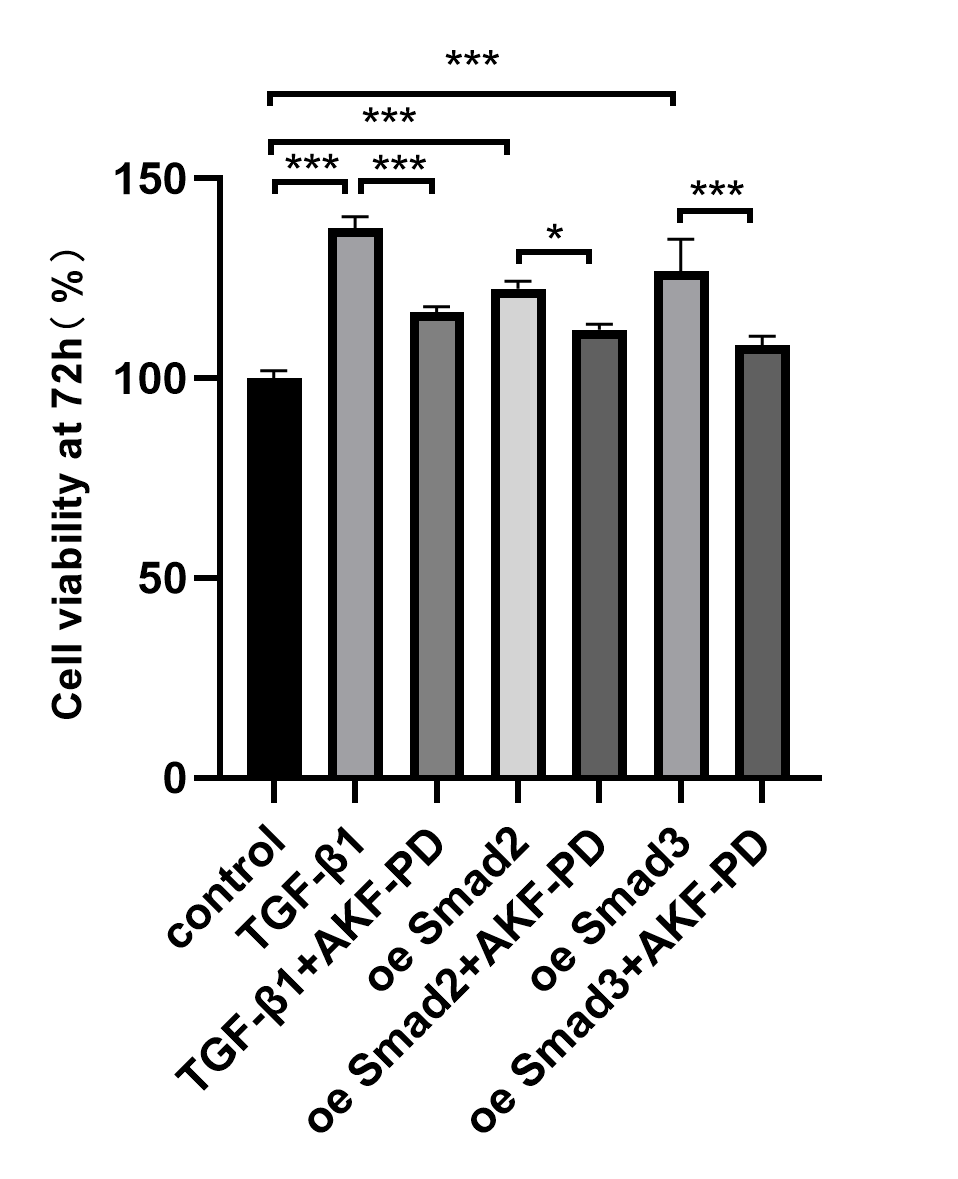

Supplement: Supplemental Information 4 [file peerj-11-16060-s004.zip › Fig4/Fig4A/Cell viability at 72h∩╝ê%∩╝ë.tif]

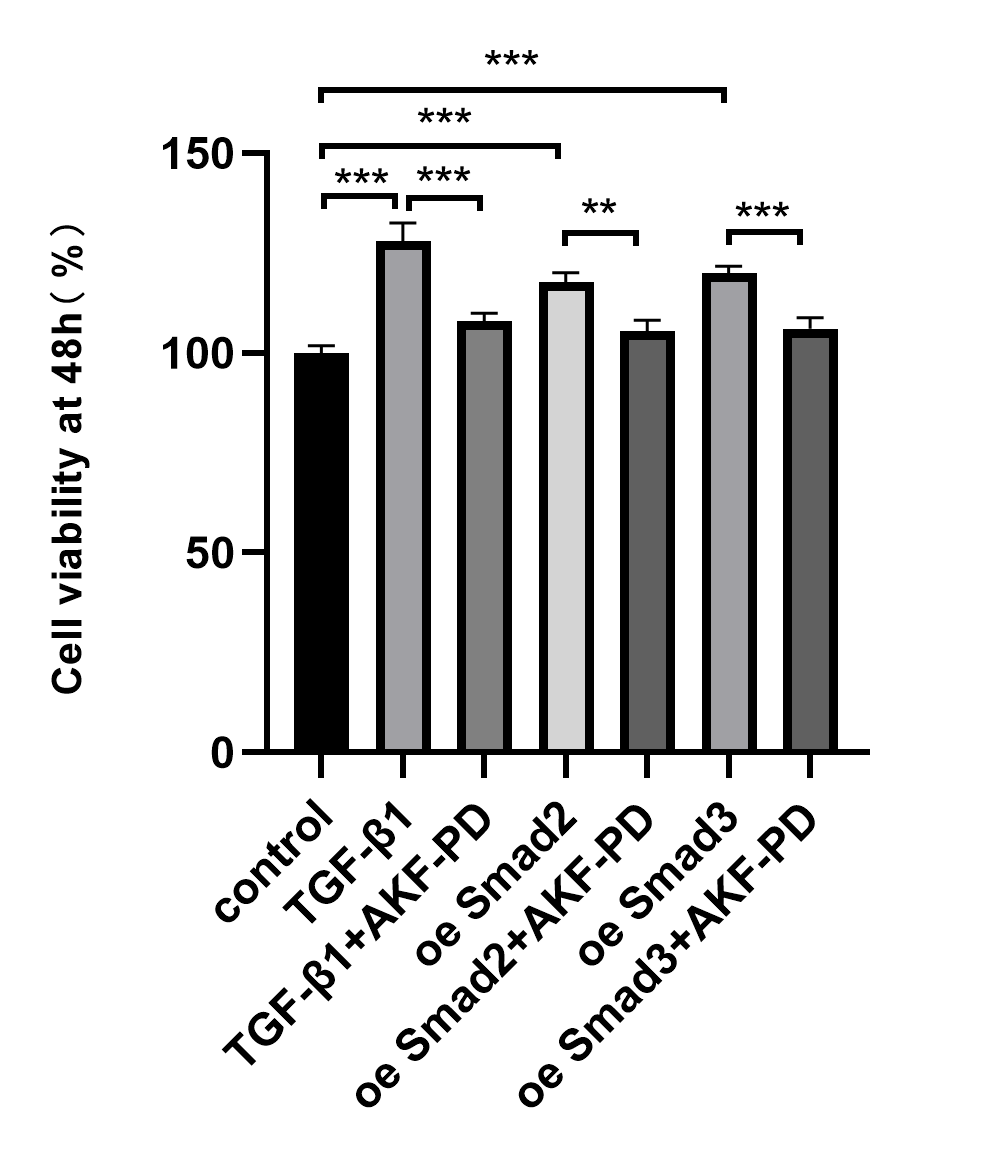

Supplement: Supplemental Information 4 [file peerj-11-16060-s004.zip › Fig4/Fig4A/Cell viability at 48h∩╝ê%∩╝ë.tif]

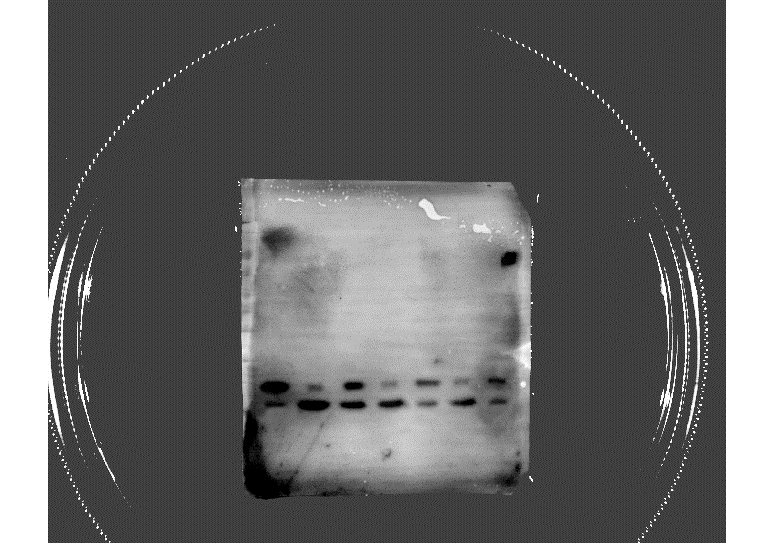

Supplement: Supplemental Information 4 [file peerj-11-16060-s004.zip › Fig4/Fig4D/LC3∩╝êΣ╕èLC3-IπÇüΣ╕ïLC3-II∩╝ë .png]

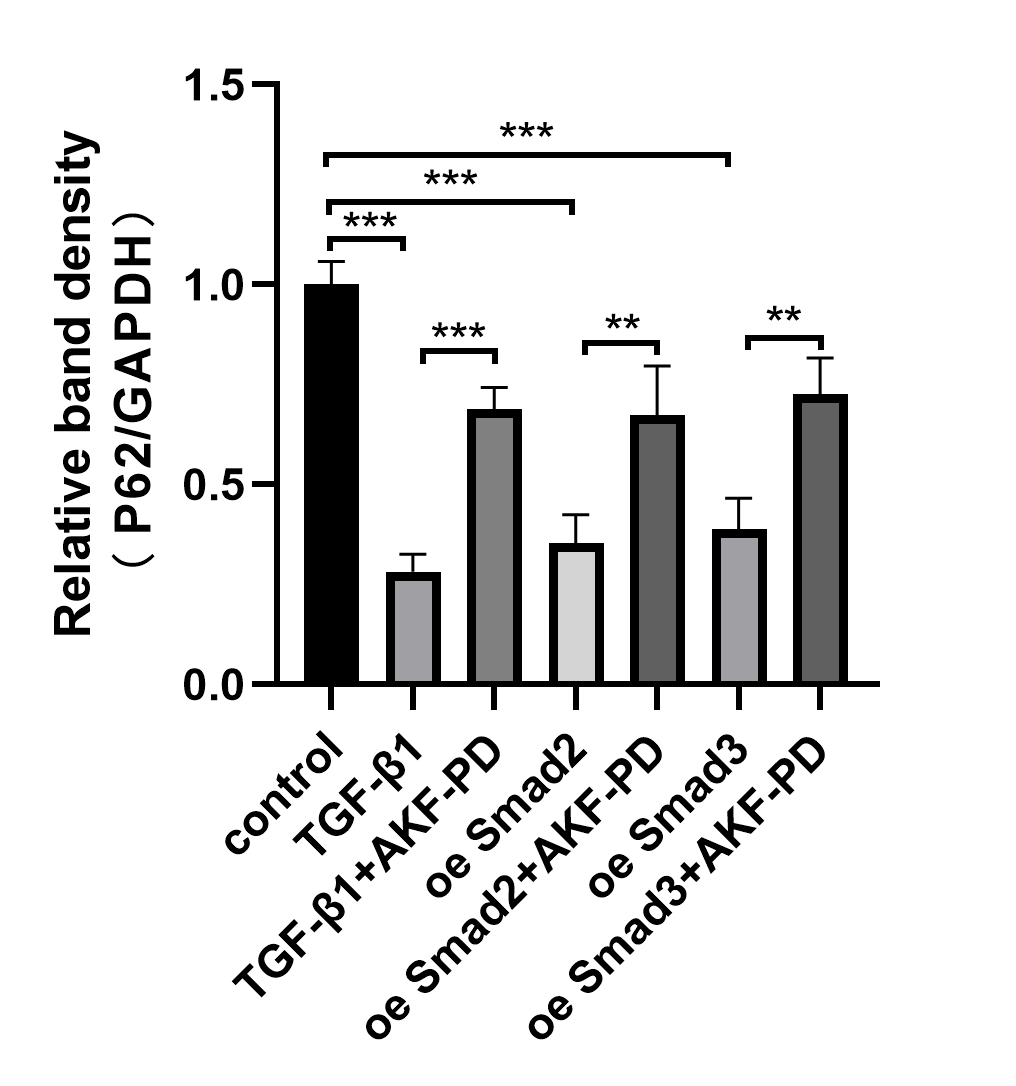

Supplement: Supplemental Information 4 [file peerj-11-16060-s004.zip › Fig4/Fig4D/Relative band density∩╝êP62_GAPDH∩╝ë.tif]

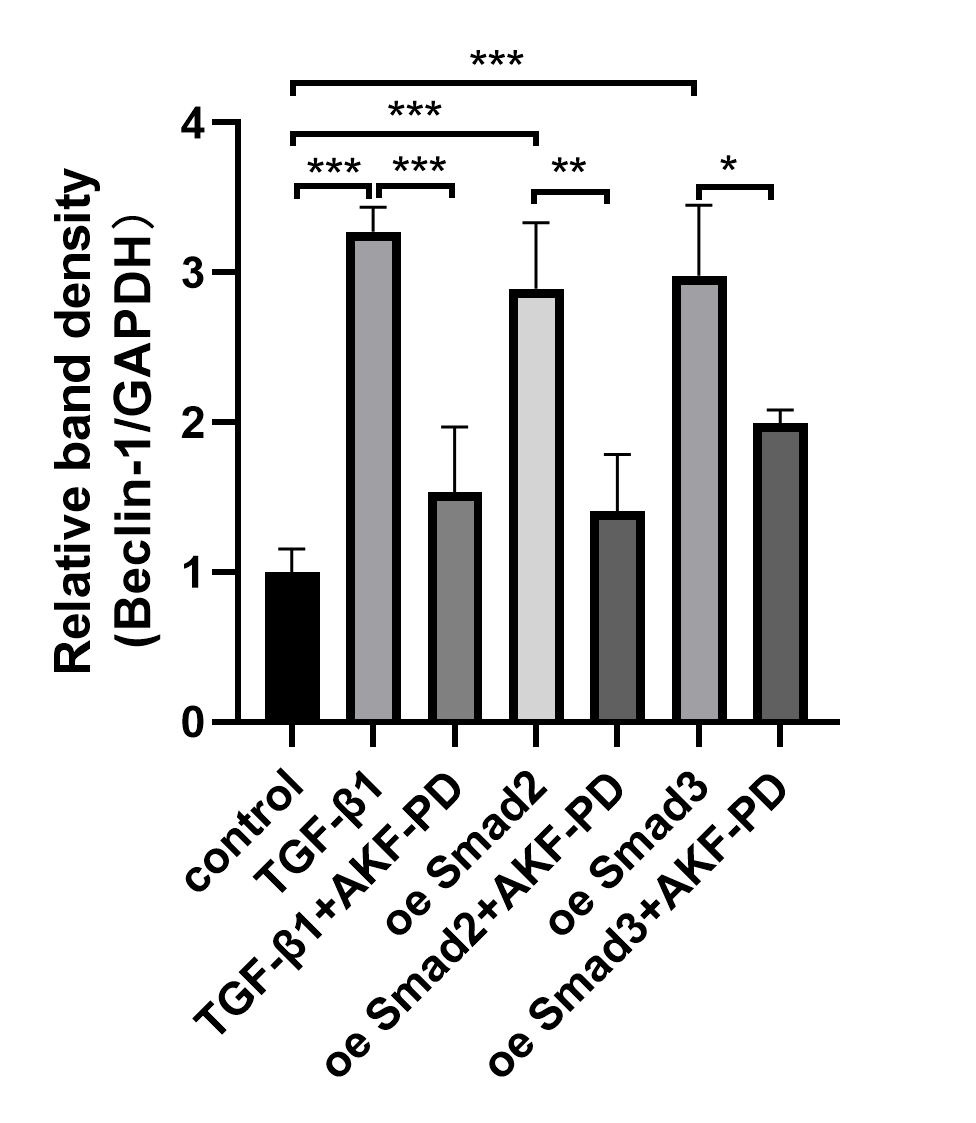

Supplement: Supplemental Information 4 [file peerj-11-16060-s004.zip › Fig4/Fig4D/Relative band density∩╝êBeclin-1_GAPDH∩╝ë.tif]

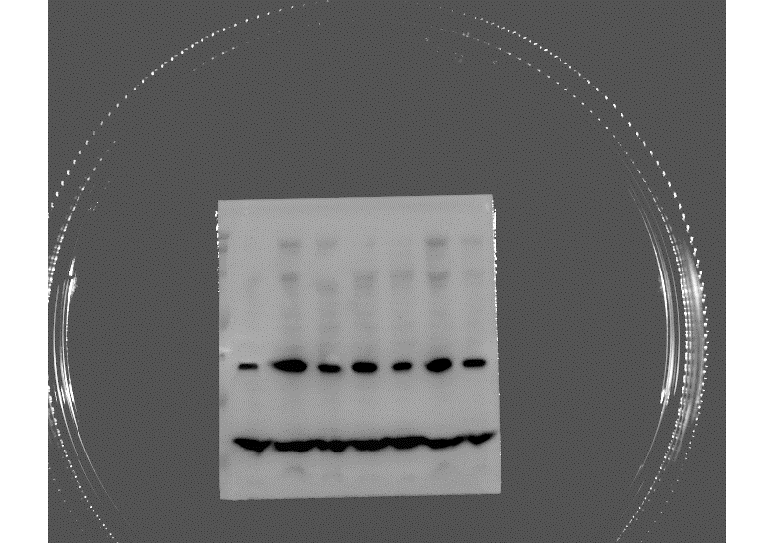

Supplement: Supplemental Information 4 [file peerj-11-16060-s004.zip › Fig4/Fig4D/Beclin-1∩╝êΣ╕è∩╝ë.png]

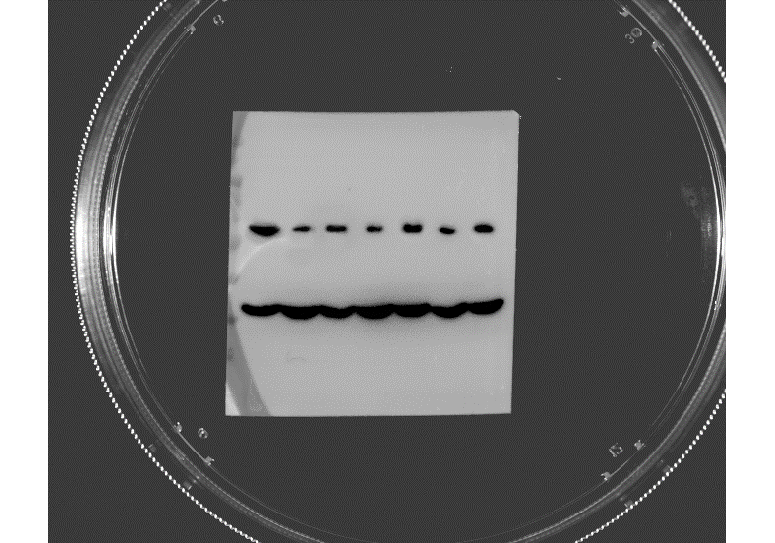

Supplement: Supplemental Information 4 [file peerj-11-16060-s004.zip › Fig4/Fig4D/P62∩╝êΣ╕èP62πÇüΣ╕ïGAPDH∩╝ë .png]

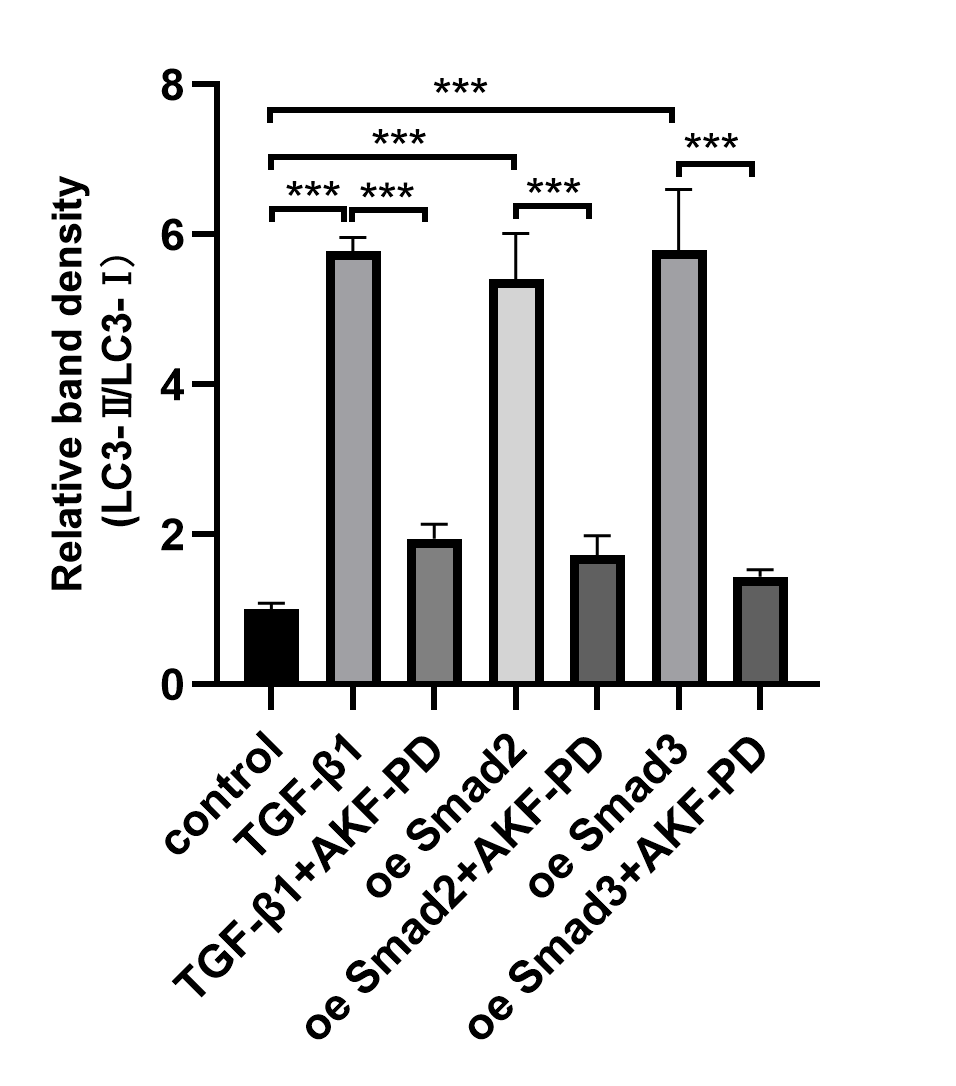

Supplement: Supplemental Information 4 [file peerj-11-16060-s004.zip › Fig4/Fig4D/Relative band density(LC3-II_LC3-Γàá∩╝ë.tif]

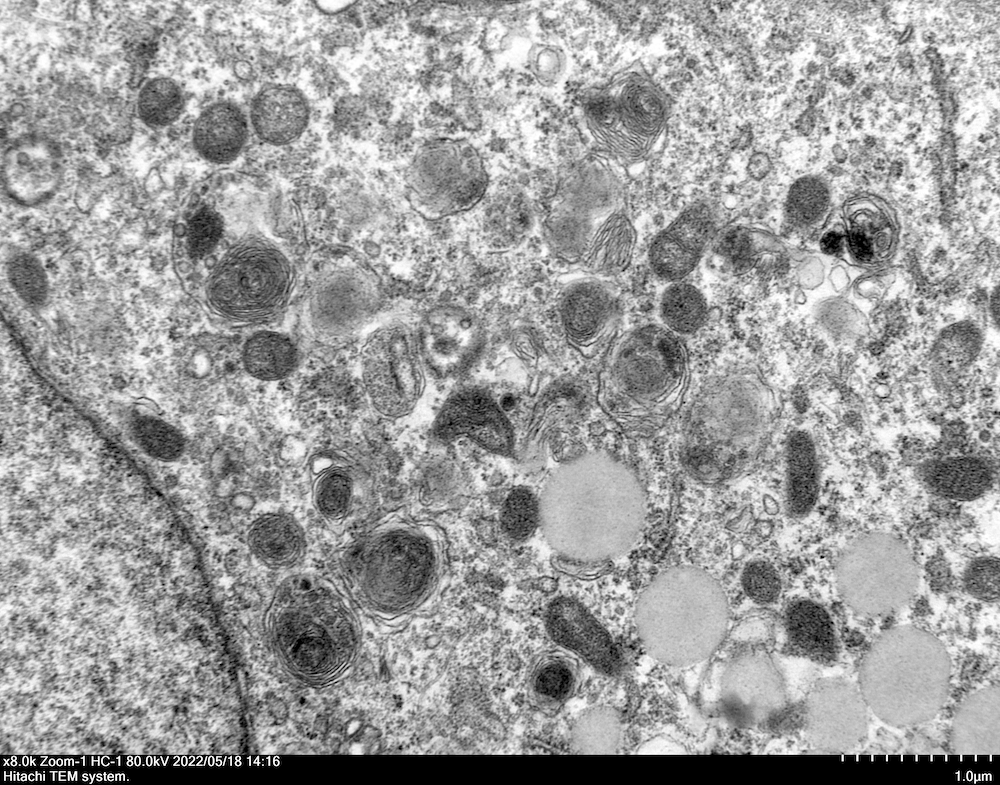

Supplement: Supplemental Information 4 [file peerj-11-16060-s004.zip › Fig4/Fig4C/TGF-╬▓1.tif]

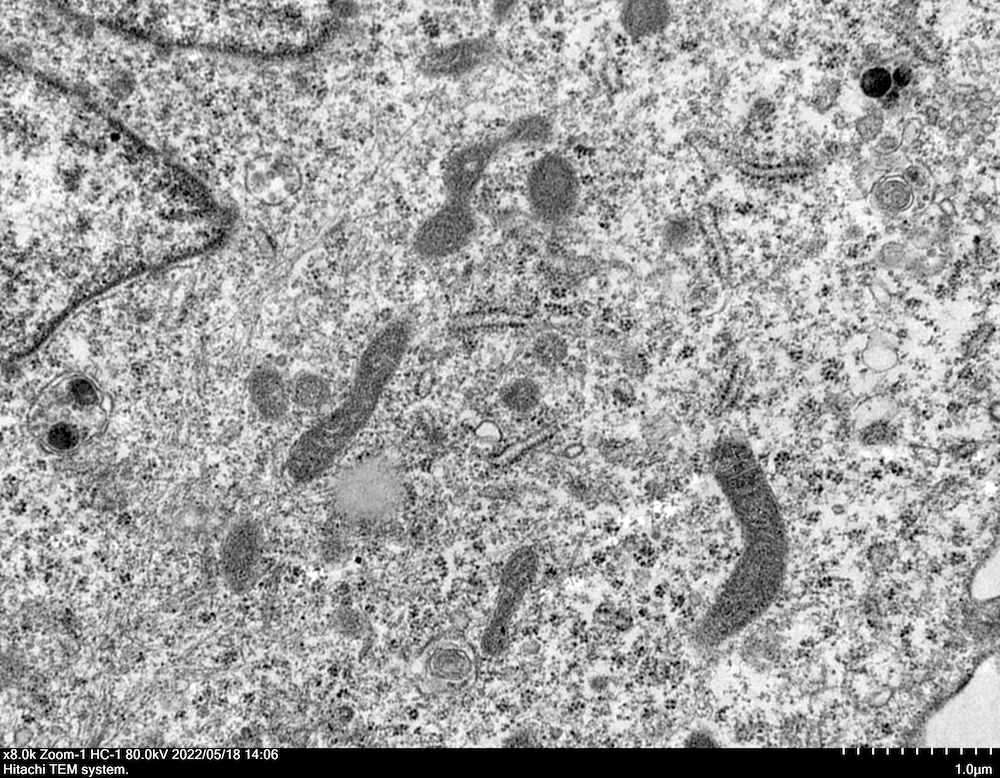

Supplement: Supplemental Information 4 [file peerj-11-16060-s004.zip › Fig4/Fig4C/Control.tif]

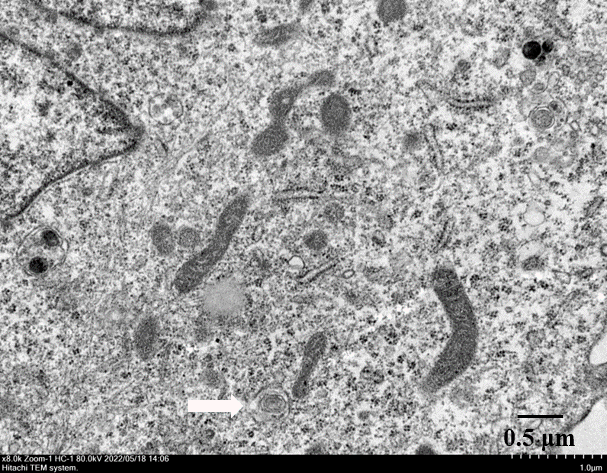

Supplement: Supplemental Information 4 [file peerj-11-16060-s004.zip › Fig4/Fig4C/Control∩╝êarrow+scale∩╝ë.png]

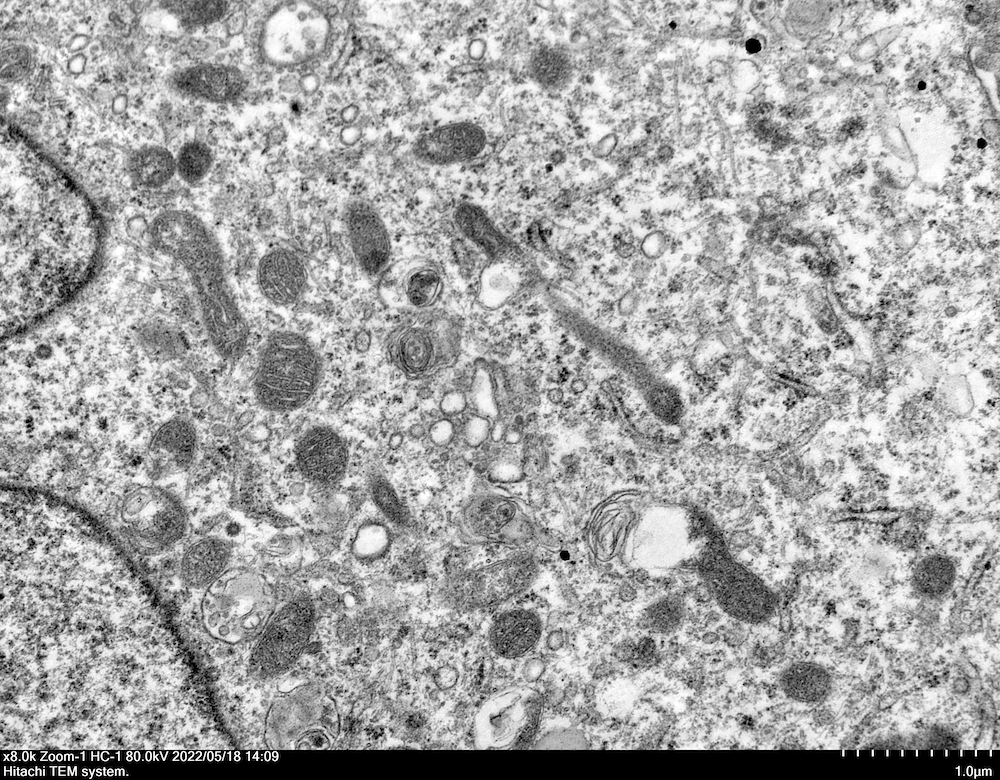

Supplement: Supplemental Information 4 [file peerj-11-16060-s004.zip › Fig4/Fig4C/TGF-╬▓1+AKF-PD.tif]

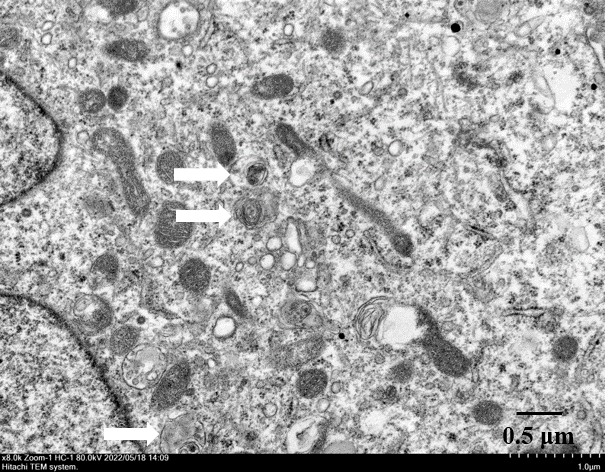

Supplement: Supplemental Information 4 [file peerj-11-16060-s004.zip › Fig4/Fig4C/TGF-╬▓1+AKF-PD∩╝êarrow+scale∩╝ë.png]

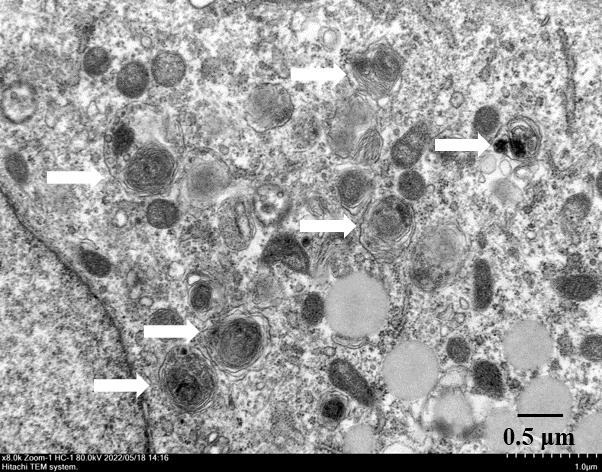

Supplement: Supplemental Information 4 [file peerj-11-16060-s004.zip › Fig4/Fig4C/TGF-╬▓1∩╝êarrow+scale∩╝ë.png]

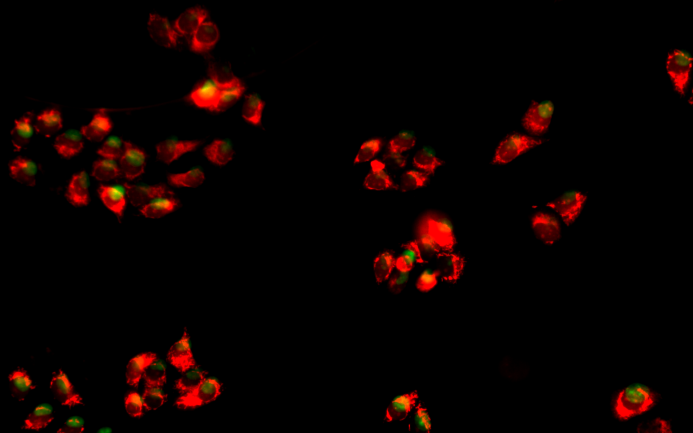

Supplement: Supplemental Information 4 [file peerj-11-16060-s004.zip › Fig4/Fig4B/TGF-╬▓1.tif]

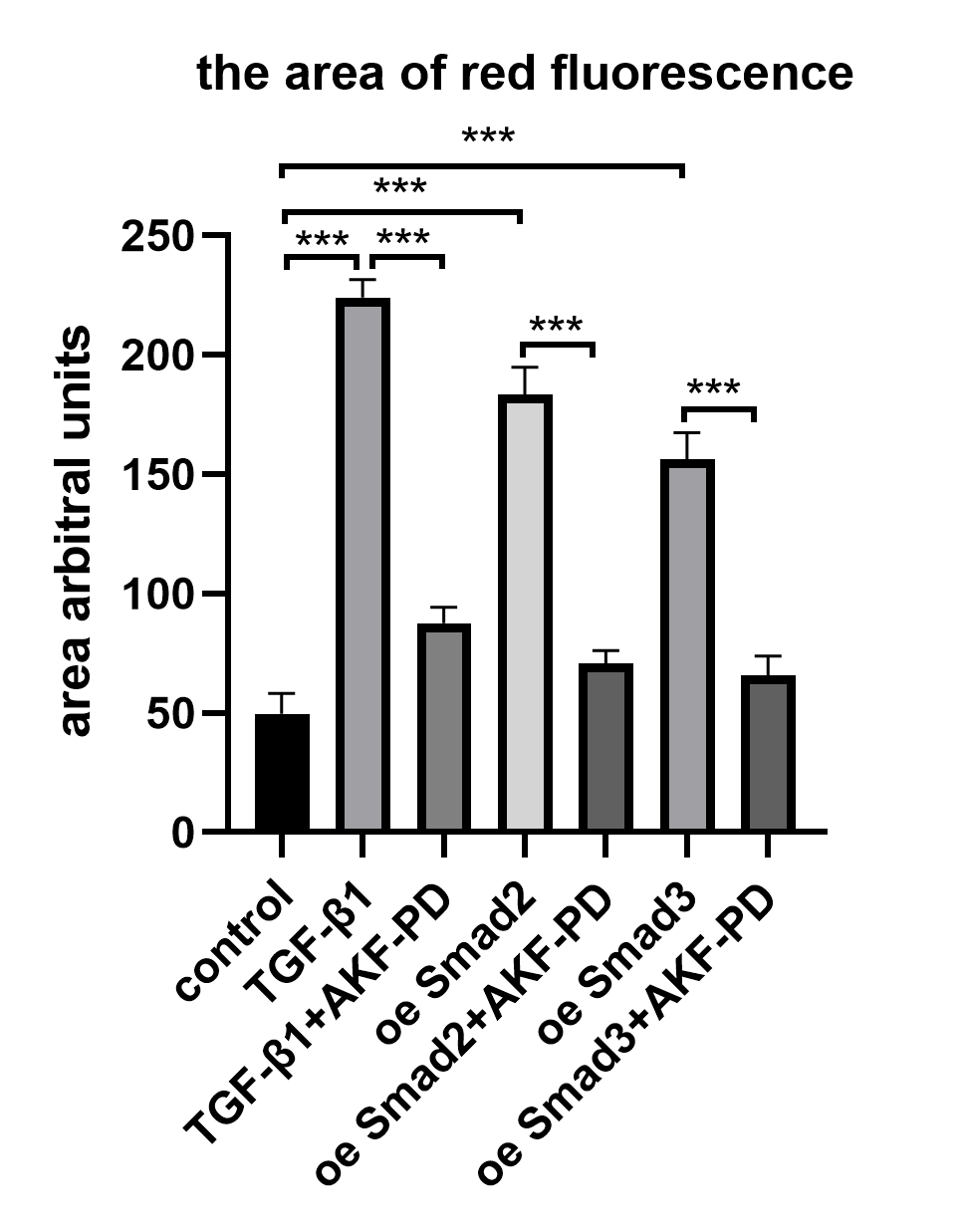

Supplement: Supplemental Information 4 [file peerj-11-16060-s004.zip › Fig4/Fig4B/area arbitral units.tif]

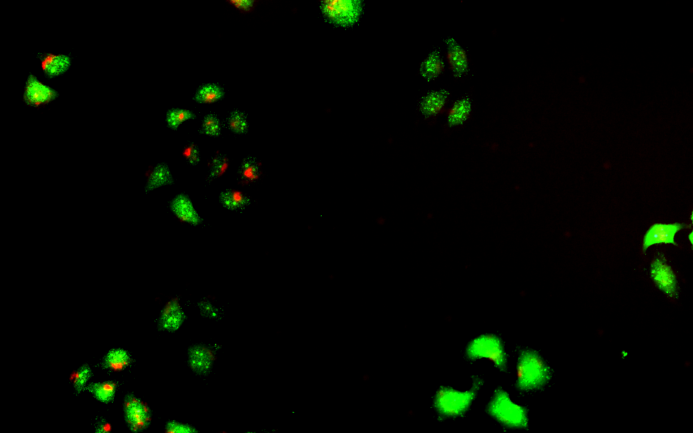

Supplement: Supplemental Information 4 [file peerj-11-16060-s004.zip › Fig4/Fig4B/control.tif]

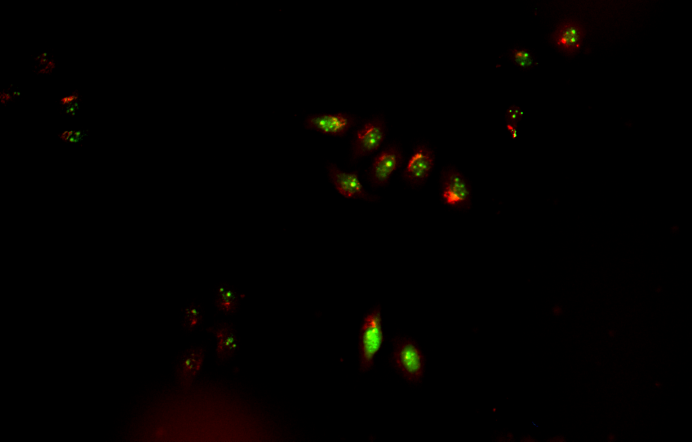

Supplement: Supplemental Information 4 [file peerj-11-16060-s004.zip › Fig4/Fig4B/oe Smad3+AKF-PD.tif]

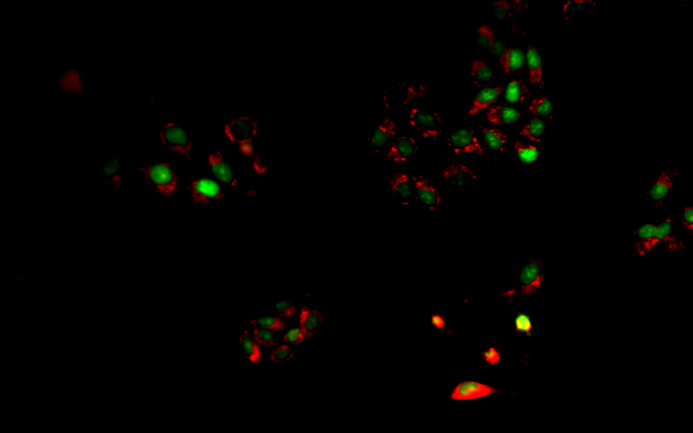

Supplement: Supplemental Information 4 [file peerj-11-16060-s004.zip › Fig4/Fig4B/TGF-╬▓1+AKF-PD.tif]

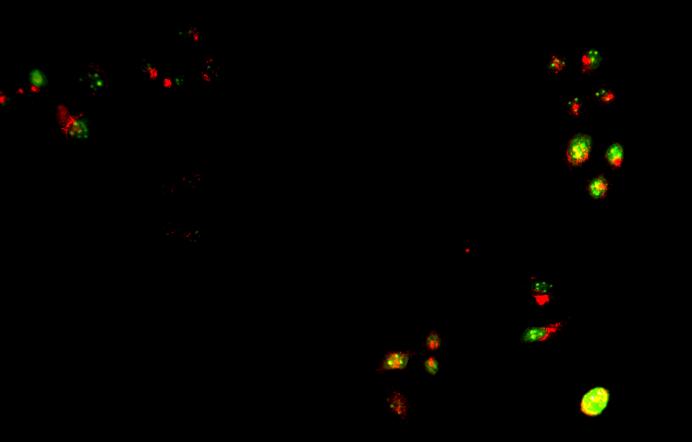

Supplement: Supplemental Information 4 [file peerj-11-16060-s004.zip › Fig4/Fig4B/oe Smad2+AKF-PD.tif]

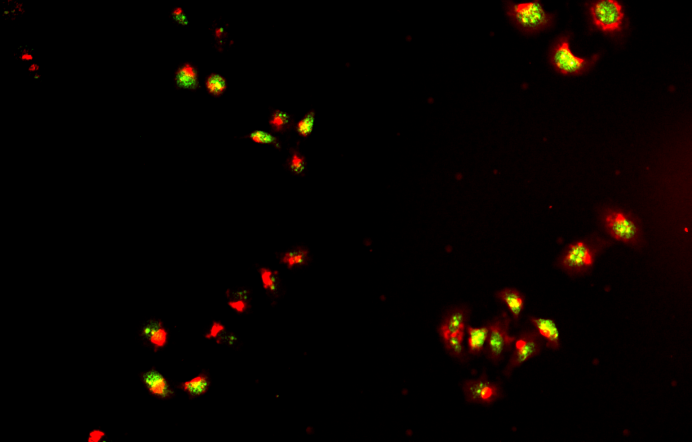

Supplement: Supplemental Information 4 [file peerj-11-16060-s004.zip › Fig4/Fig4B/oe Smad3.tif]

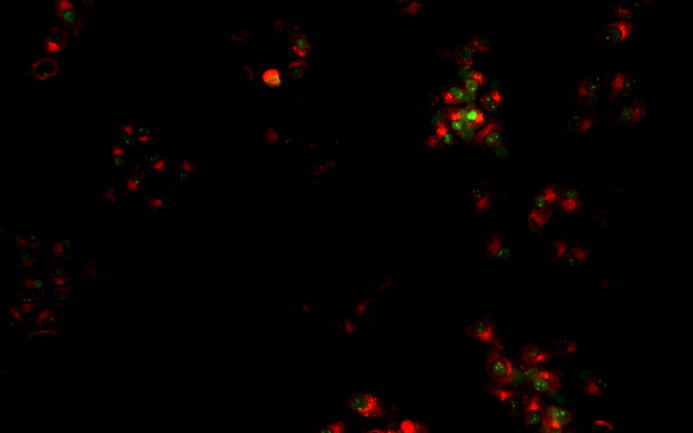

Supplement: Supplemental Information 4 [file peerj-11-16060-s004.zip › Fig4/Fig4B/oe Smad2.tif]

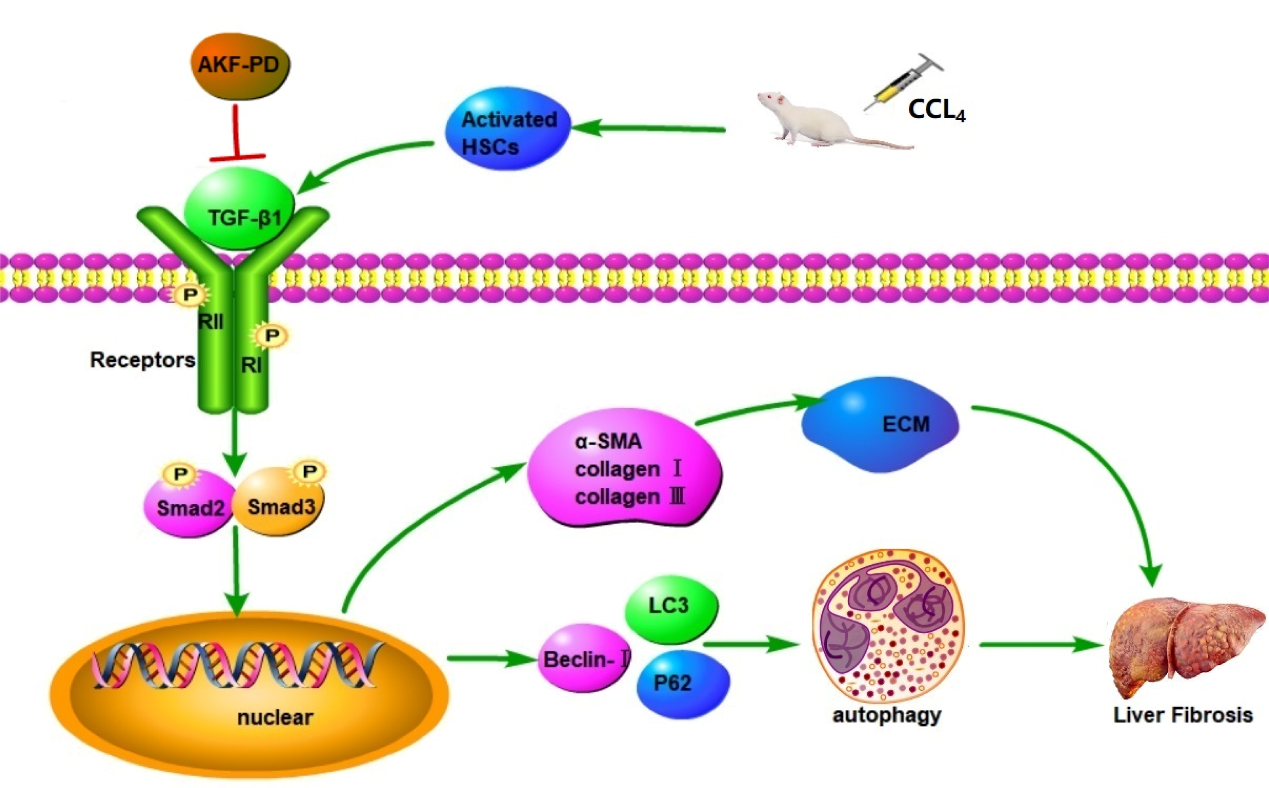

Supplement: Supplemental Information 5 [file peerj-11-16060-s005.zip › Fig5/Fig5.tif]
